# Supplementary material for: Structure-conserving spontaneous transformations between nanoparticles
Source: Nat Commun. 2016 Nov 10;7:13447. doi: 10.1038/ncomms13447 (PMC5110647; doi:10.1038/ncomms13447)
Supplement: Supplementary Information — Supplementary Figures 1-21, Supplementary Tables 1-8, Supplementary Note 7 and Supplementary References. [file ncomms13447-s1.pdf]

Supplementary Figures

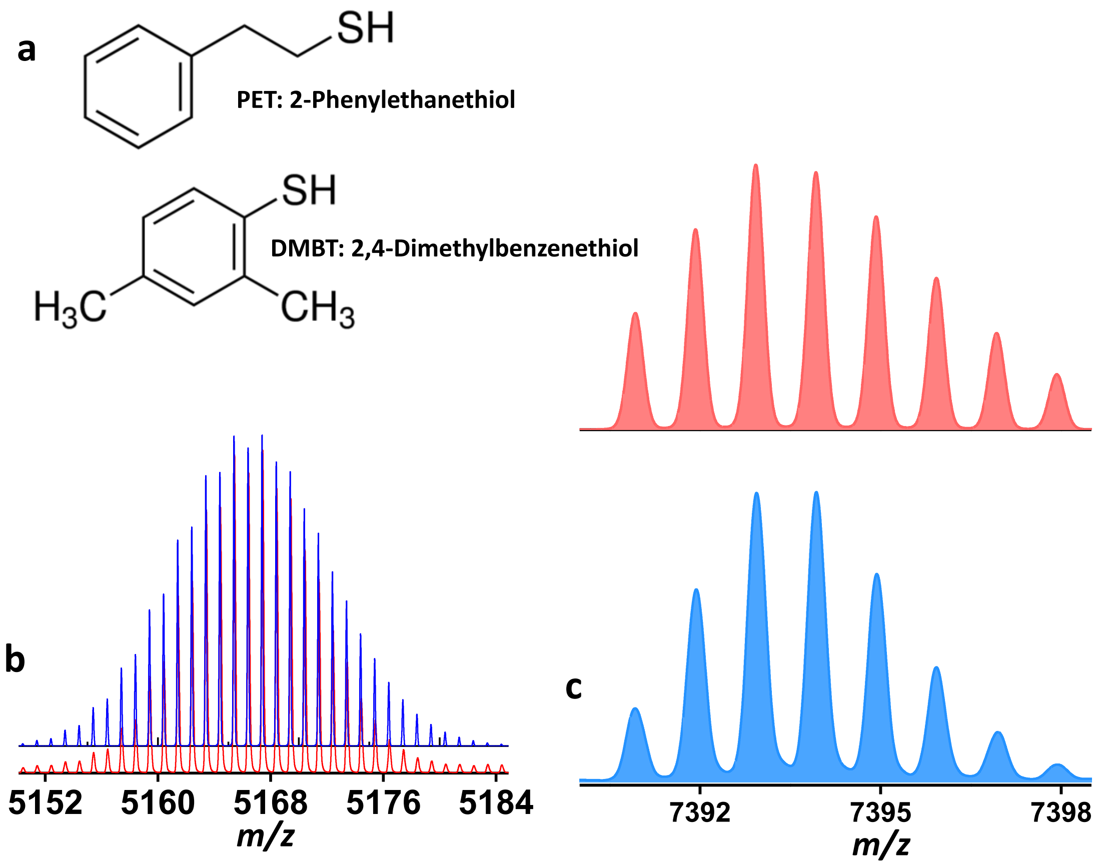

Supplementary Figure 1. Molecular structures of the ligands and the isotope patterns of the clusters Molecular structures of ligands 2-phenylethanethiol (PET) and 2,4-dimethylbenzenethiol (DMBT) (a), theoretical (red) and experimental (blue) isotope patterns for $Ag_{25}(DMBT)_{18}$ (b) and $Au_{25}(PET)_{18}$ (c). These data illustrate the resolution and the spectral quality available for these systems.

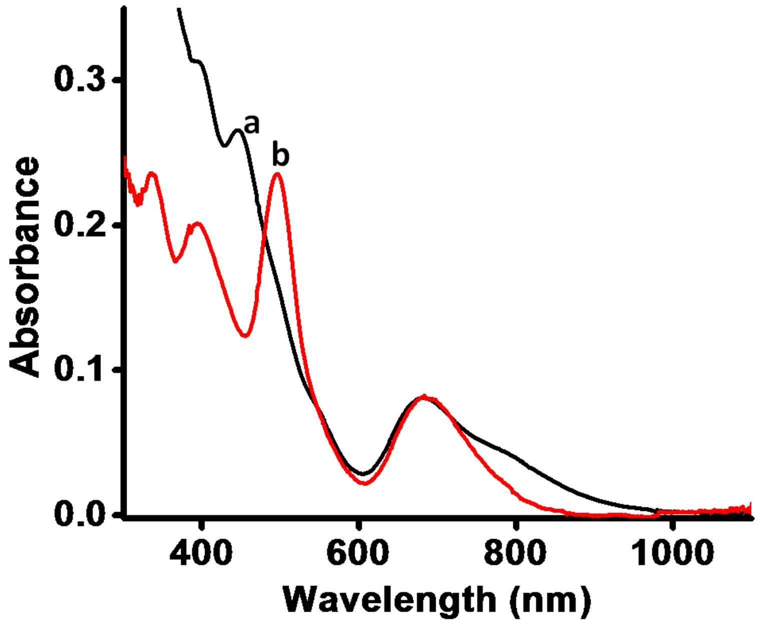

Supplementary Figure 2. UV/Vis absorption features of clusters Characteristic UV/Vis absorption features of $Ag_{25}(DMBT)_{18}$ (a, black) and $Au_{25}(PET)_{18}$ (b, red). PET is 2-phenylethanethiol and DMBT is 2,4-dimethylbenzenethiol.

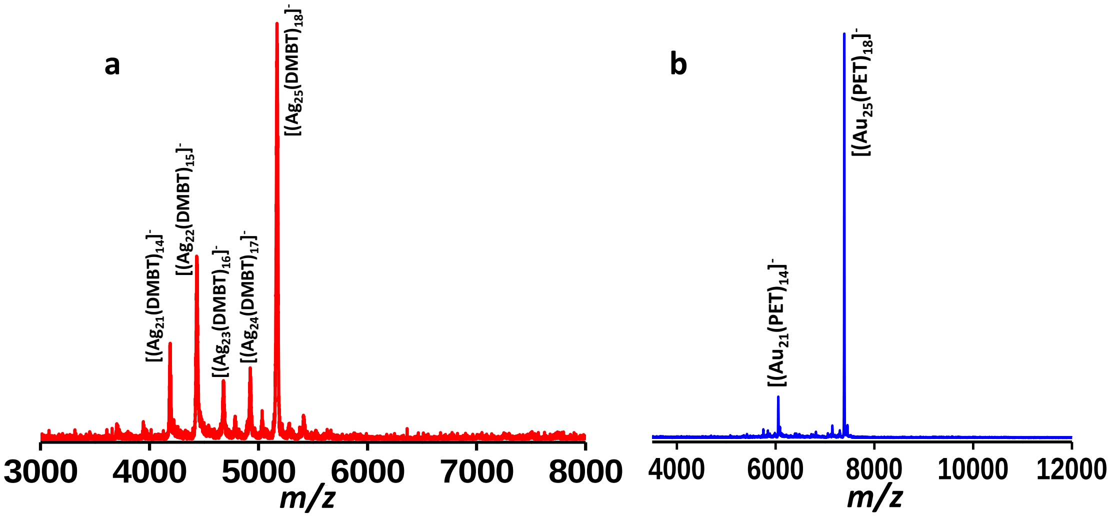

Supplementary Figure 3. Matrix assisted laser desorption ionization mass spectra of the clusters Negative ion mode Matrix assisted laser desorption ionization mass spectra (MALDI MS) of $Ag_{25}(DMBT)_{18}$ (a) and $Au_{25}(PET)_{18}$ (b). Characteristic fragment peaks are shown in each

spectrum. PET is 2-phenylethanethiol and DMBT is 2,4-dimethylbenzenethiol. The loss of Ag-DMBT (in $Ag_{25}(DMBT)_{18}$) and that of $Au_4(PET)_4$ (in $Au_{25}(PET)_{18}$) are the expected fragmentation patterns of these clusters.

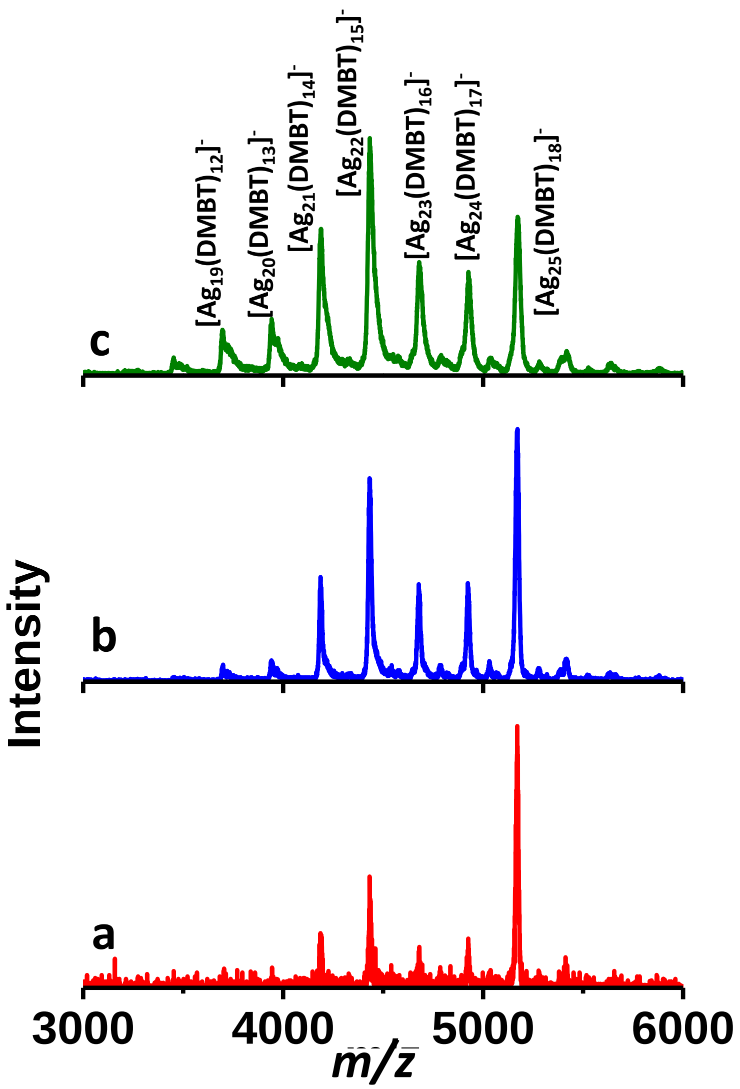

Supplementary Figure 4. Matrix assisted laser desorption ionization mass spectra (MALDI MS) of $Ag_{25}(DMBT)_{18}$ at various laser fluences MALDI MS spectra of $Ag_{25}(DMBT)_{18}$ at laser fluences of 1150 (a), 1350 (b) and 1450 (c). With increasing fluence, more fragmentation is observed. Laser fluence is shown as given by the instrument.

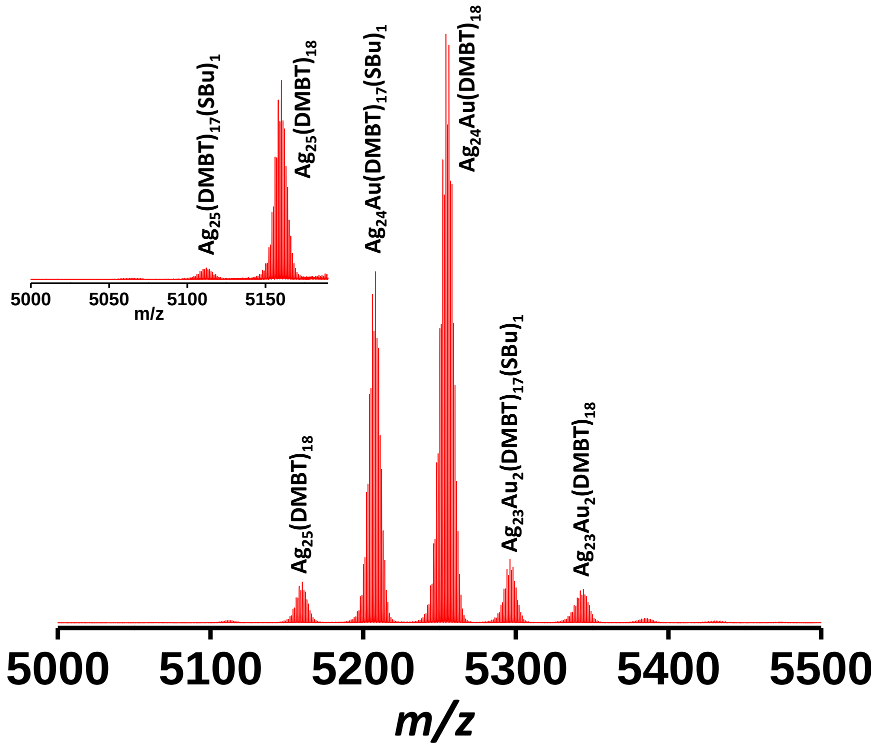

Supplementary Figure 5. Electrospray ionization mass spectra (ESI MS) showing ligand and metal-ligand fragment exchanges ESI MS spectra showing the exchange products resulting from Ag-Au, (DMBT)-(SBu) and (Ag-DMBT)-(Au-SBu) exchanges between $Ag_{25}(DMBT)_{18}$ and $Au_{25}(SBu)_{18}$. The inset shows that parent $Ag_{25}(SR)_{18}$ undergoes DMBT-SBu exchange also. PET is 2-phenylethanethiol and DMBT is 2,4-dimethylbenzenethiol.

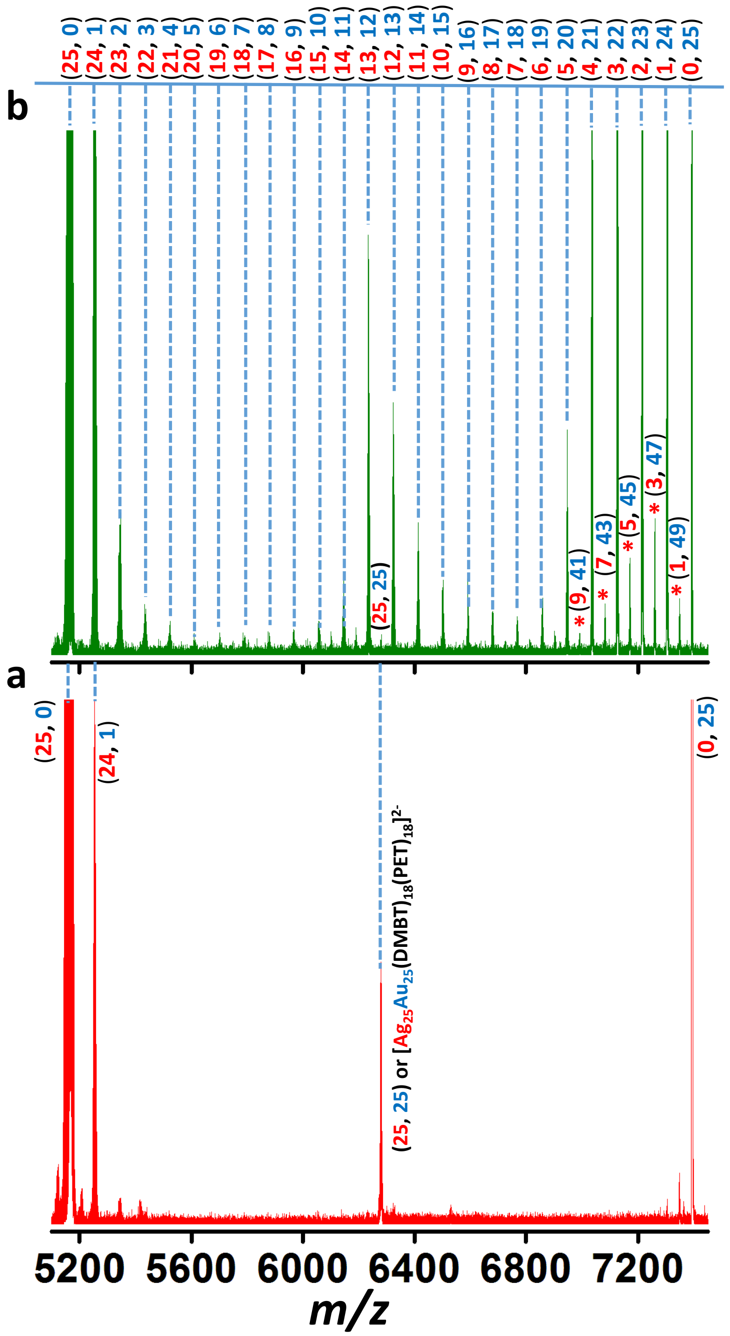

Supplementary Figure 6. Evolution of alloy clusters from the dianionic adduct Electrospray ionization mass spectra (ESI MS) of a reaction mixture at the $Ag_{25}(DMBT)_{18}$:$Au_{25}(PET)_{18}$ molar ratio of 0.3:1.0 measured (a) within 2 min after mixing and (b) 5 minutes after mixing. Panel a shows the peak due to $[Ag_{25}Au_{25}(DMBT)_{18}(PET)_{18}]^{2-}$, the adduct formed between $[Ag_{25}(DMBT)_{18}]^{-}$ and $[Au_{25}(PET)_{18}]^{-}$. PET is 2-phenylethanethiol and DMBT is 2,4-dimethylbenzenethiol. Panel (b) shows that this peak disappeared almost completely within 5

min after mixing of the clusters and monoanionic alloy clusters are formed. Numbers in the parentheses of the labels of main peaks in (a) and (b) correspond to the general formula, $[Ag_mAu_n(SR)_{18}]^-$ (m+n=25) (for monoanionic alloys). Numbers in the parentheses of the labels of the peaks marked with * in panel (b) correspond to the general formula, $[Ag_mAu_n(SR)_{36}]^{2-}$ (m+n=50) (for the dianionic adducts). Some of these ions exist in (a) also.

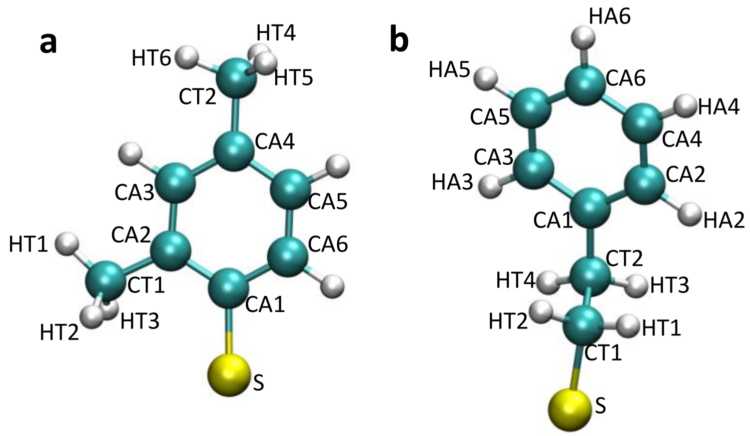

Supplementary Figure 7. Molecular structures and the labeling of the different types of atoms in (a) 2,4-dimethylbenzenethiol (DMBT) and (b) 2-phenylethanethiol (PET) ligands.

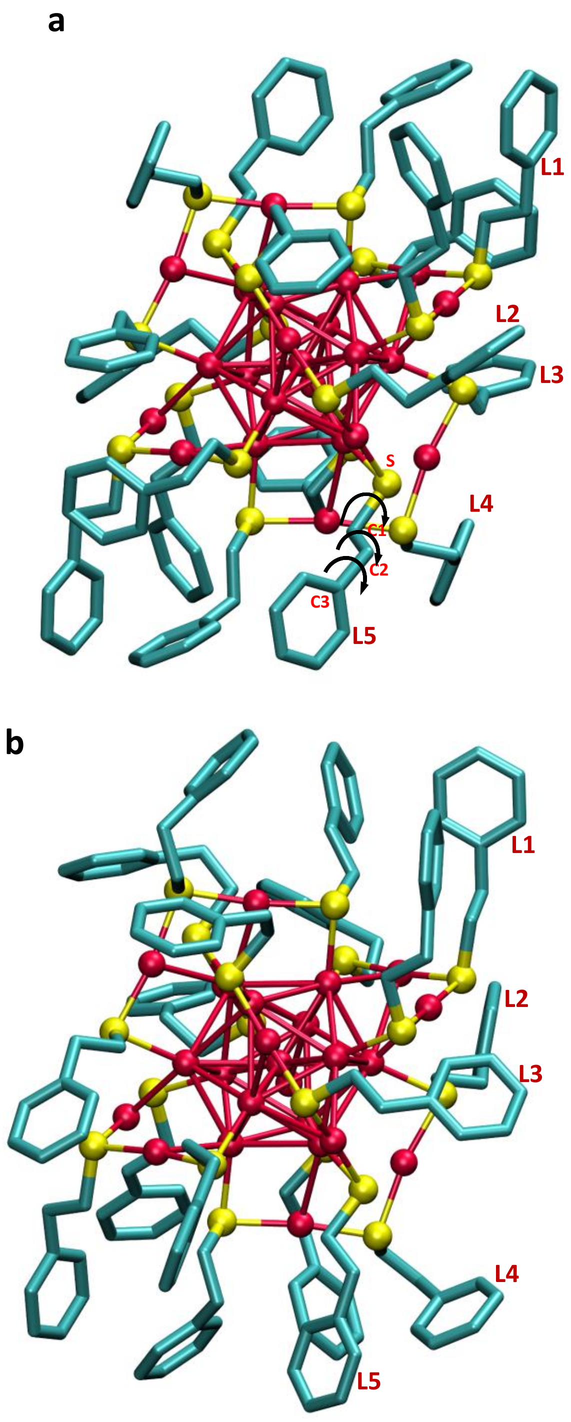

Supplementary Figure 8. Initial and final configurations of $Au_{25}(PET)_{18}$ in the force-field global minimum of adduct Initial (a) and after (b) configuration of $Au_{25}(PET)_{18}$. PET (2-phenylethanethiol) ligands which have undergone rotations are marked in each picture with corresponding directions of rotations marked in them.

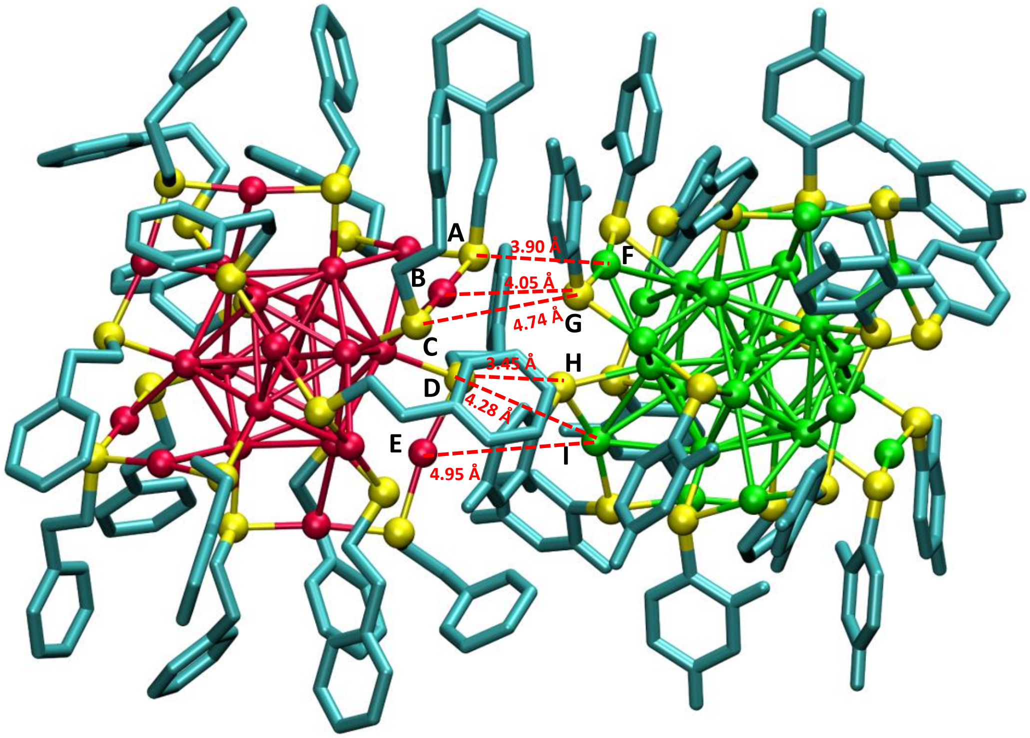

Supplementary Figure 9. Minimum energy configuration of the clusters in the force-field global minimum of adduct. The $Au_{25}(PET)_{18}$-$Ag_{25}(DMBT)_{18}$ (**II**-**I**) force-field global minimum of adduct from a molecular docking simulation with **II** on the left and **I** on the right. Dashed lines show the shortest distances found between atoms in the staples of the two clusters which are marked with letters A to E on **II** and F to I on **I**. Distances between the pairs of metal and sulfur atoms labelled are AF ($S_b(Au_{25})$-Ag)=3.90 Å, BG (Au-$S_b(Ag_{25})$)=4.05 Å, CG ($S_{nb}(Au_{25})$-$S_b(Ag_{25})$)=4.74 Å, DH ($S_{nb}(Au_{25})$-$S_{nb}(Ag_{25})$)=3.45 Å, DI ($S_{nb}(Au_{25})$-Ag)=4.28 Å and EI (Au-Ag)=4.95 Å. The brackets after the pair of letters gives details of the element types and the bridging and non-bridging sulfur positions on staples that are denoted by the subscripts b and nb, respectively. The hydrogen atoms are omitted from the ligands for clarity. Color code for the atoms: Au (red), Ag (green), S (yellow), C (blue). PET is 2-phenylethanethiol and DMBT is 2,4-dimethylbenzenethiol.

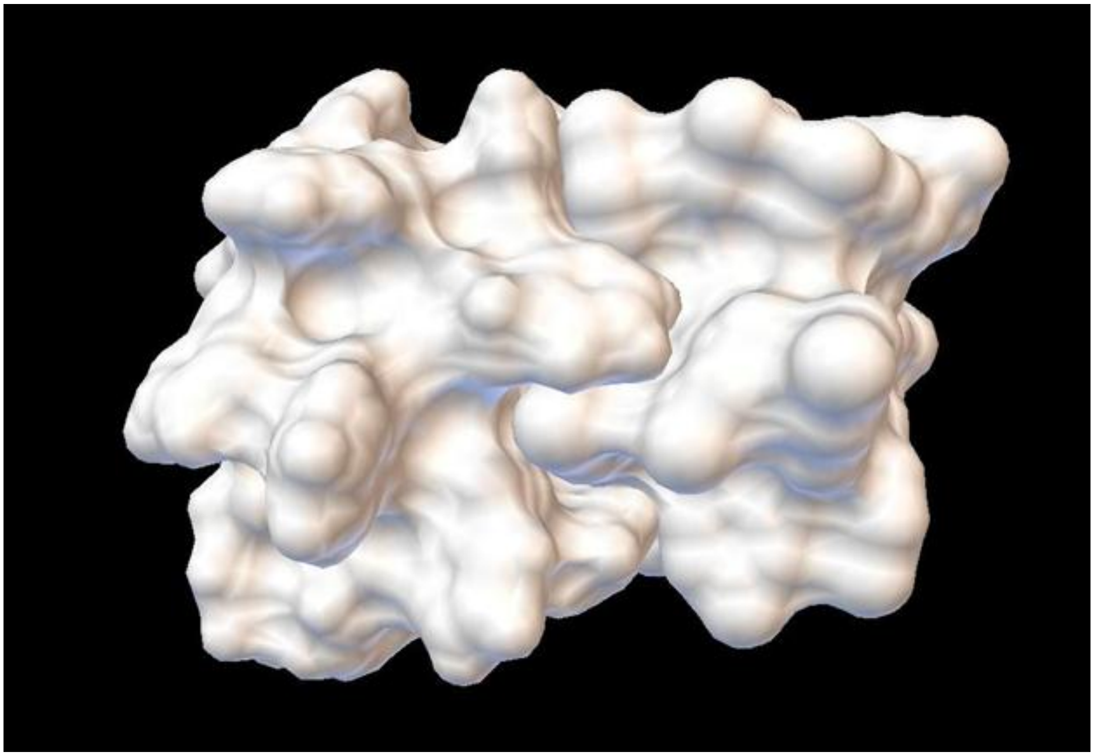

Supplementary Figure 10. Molecular surfaces of the clusters in the force-field global minimum of adduct The relative orientation of the $Ag_{25}(DMBT)_{18}$ (right) and $Au_{25}(PET)_{18}$ (left) in the minimum energy configuration (shown in Figure 3 and Supplementary Fig. 9) depicting that the overlap between the molecular surface or envelope of the ligands of the two clusters has maximal area of contact. PET is 2-phenylethanethiol and DMBT is 2,4-dimethylbenzenethiol.

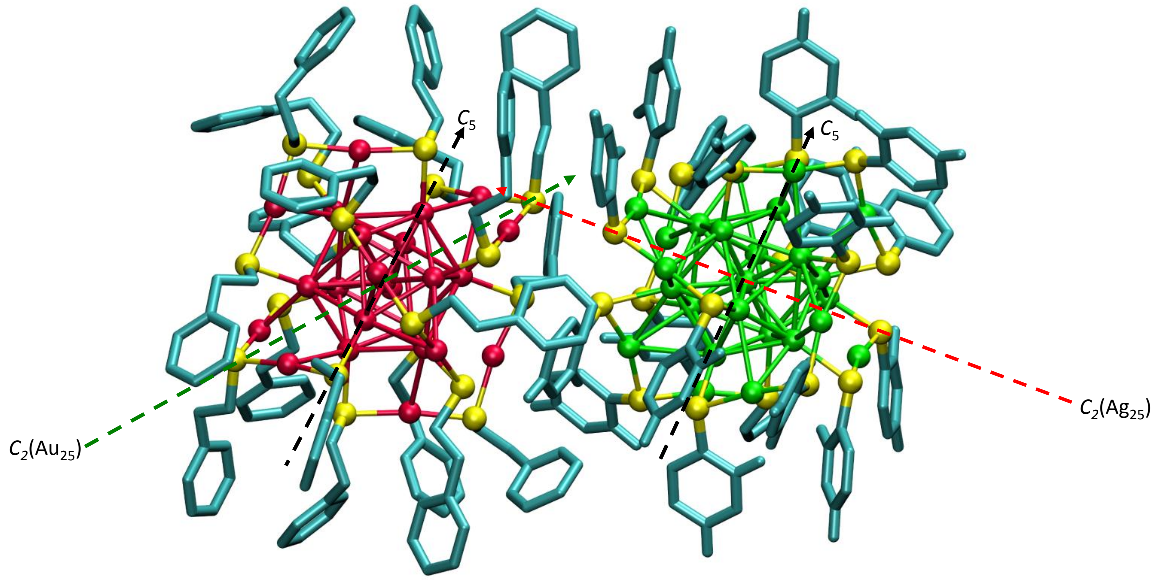

Supplementary Figure 11. Orientation of the clusters in their force-field global minimum of adduct The orientation of the $Au_{25}(PET)_{18}$ and $Ag_{25}(DMBT)_{18}$ in their docked configuration is shown with respect to their relevant symmetry axes. One of the C_2 axes of $Au_{25}(PET)_{18}$ is shown by the green dashed arrow and one of the C_2 axes of $Ag_{25}(DMBT)_{18}$ is shown by the red dashed arrow. The two C_5 axes shown by the vertical black dashed arrows are nearly parallel to each other. PET is 2-phenylethanethiol and DMBT is 2,4-dimethylbenzenethiol.

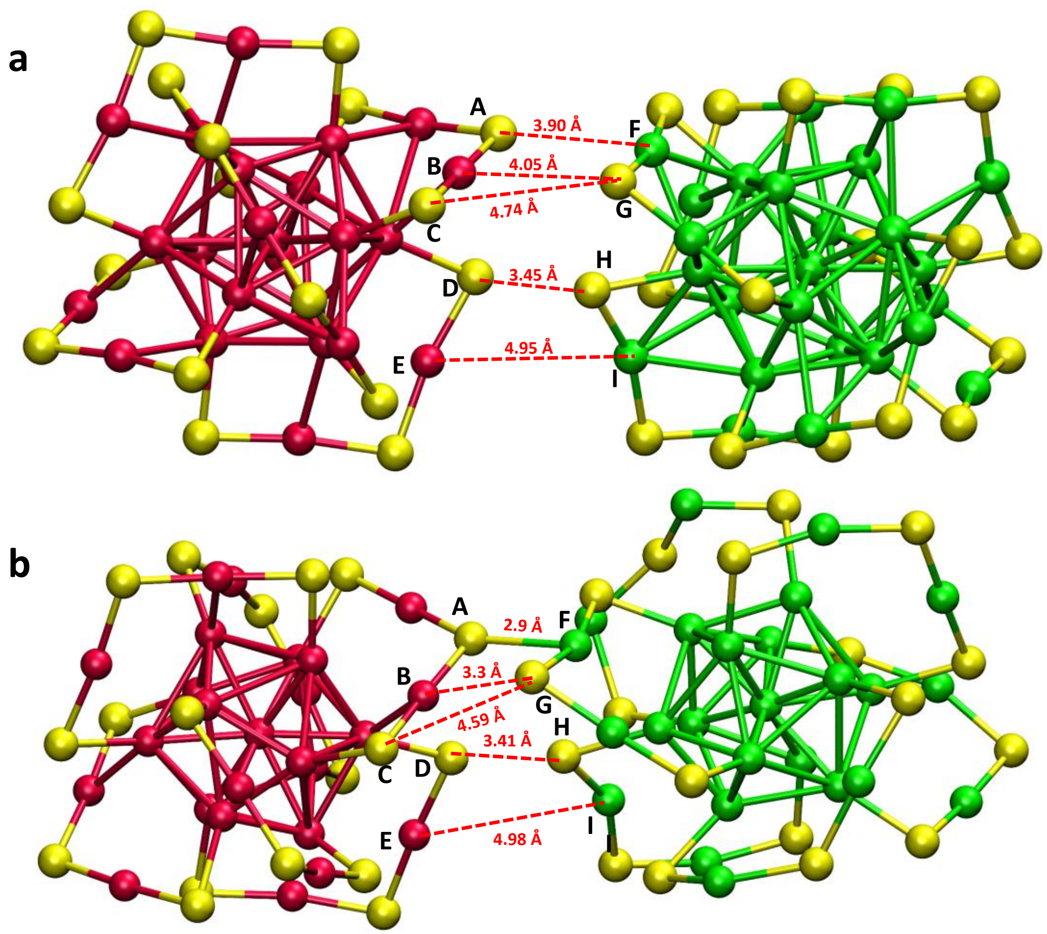

Supplementary Figure 12. Comparison of the distances between the closest metal atoms and the sulfur atoms at the interface of $Au_{25}(PET)_{18}$ (left) and $Ag_{25}(DMBT)_{18}$ (right) in the force-field global minimum of adduct (a) and its DFT-optimized geometry (b). Ligands are omitted for clarity. Color code: red (Au), green (Ag) and yellow (sulfur). PET is 2-phenylethanethiol and DMBT is 2,4-dimethylbenzenethiol.

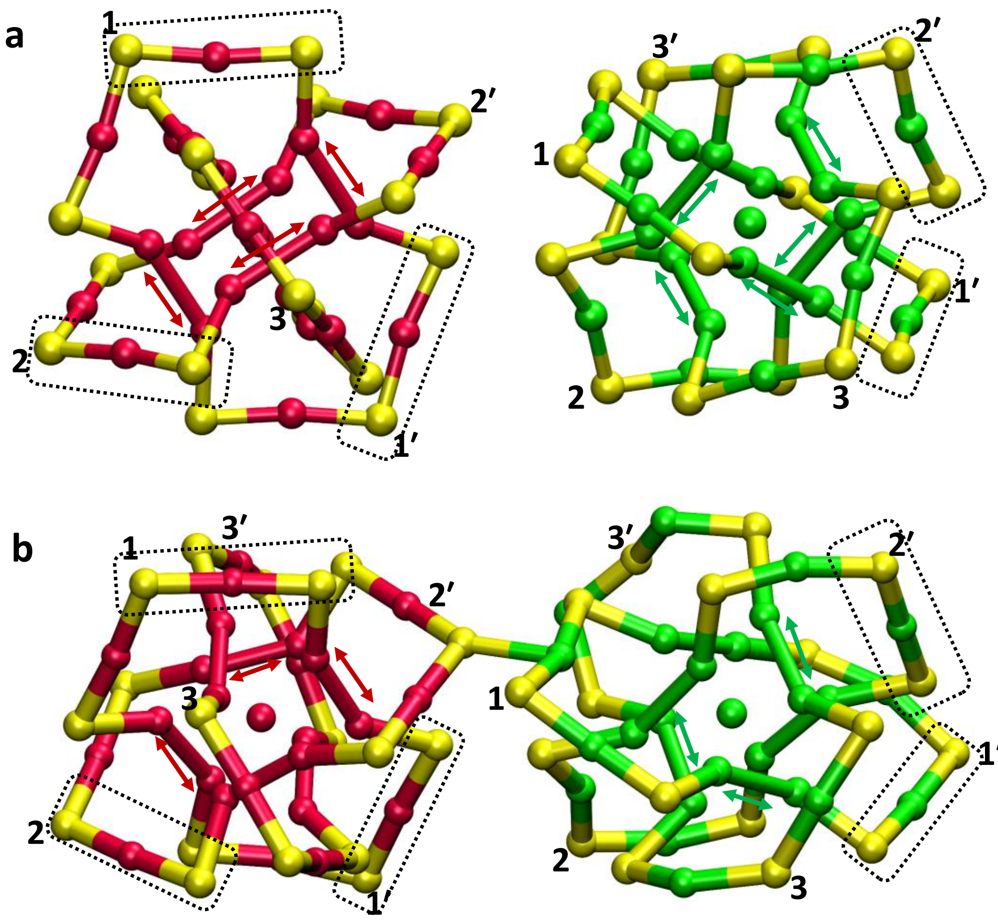

Supplementary Figure 13. Comparison of the changes in the $M_2(SR)_3$ staples (labeled 1-1′, 2-2′ and 3-3′) of $Au_{25}(PET)_{18}$ (left) and $Ag_{25}(DMBT)_{18}$ (right) (ligands omitted for clarity) in their force-field global minimum of adduct (a) and its DFT-optimized geometry (b). Color codes: red (Au), green (Ag) and yellow (S). In (a) and (b), the opposite pairs of $M_2(SR)_3$ staples labeled 1-1′, 2-2′ and 3-3′ are joined by the shorter M-M bonds (labeled by red and green arrows) of the icosahedron to form $M_8(SR)_6$ rings. The other metal atoms in the icosahedron and the longer M-M bonds are omitted for clarity. Please refer to Supplementary Ref. 1 below for the description of the shorter and the longer bonds. Comparison of 1-1′, 2-2′ and 3-3′ in each of these adducts show that the some of the S-M-S fragments (marked in dotted rectangles) of the staples became more linear in b compared to the V-shape in (a). PET is 2-phenylethanethiol and DMBT is 2,4-dimethylbenzenethiol.

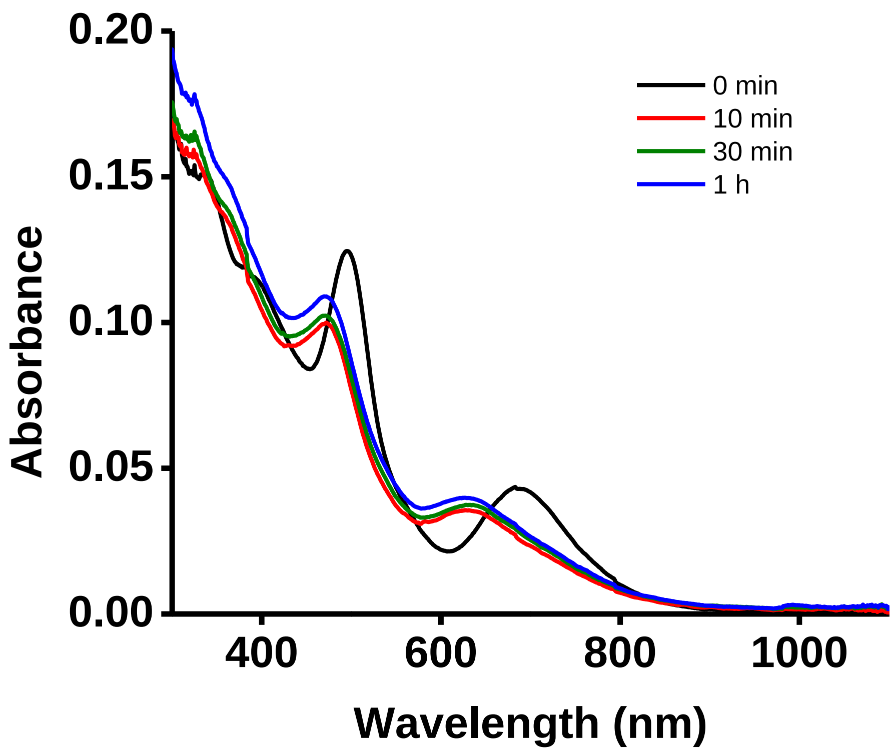

Supplementary Figure 14. Time-dependent change in the UV/Vis absorption spectra during the reaction between $Ag_{25}(DMBT)_{18}$ and $Au_{25}(PET)_{18}$ at a molar ratio (Ag_{25}:Au_{25}) of 6.6:1.0. PET is 2-phenylethanethiol and DMBT is 2,4-dimethylbenzenethiol.

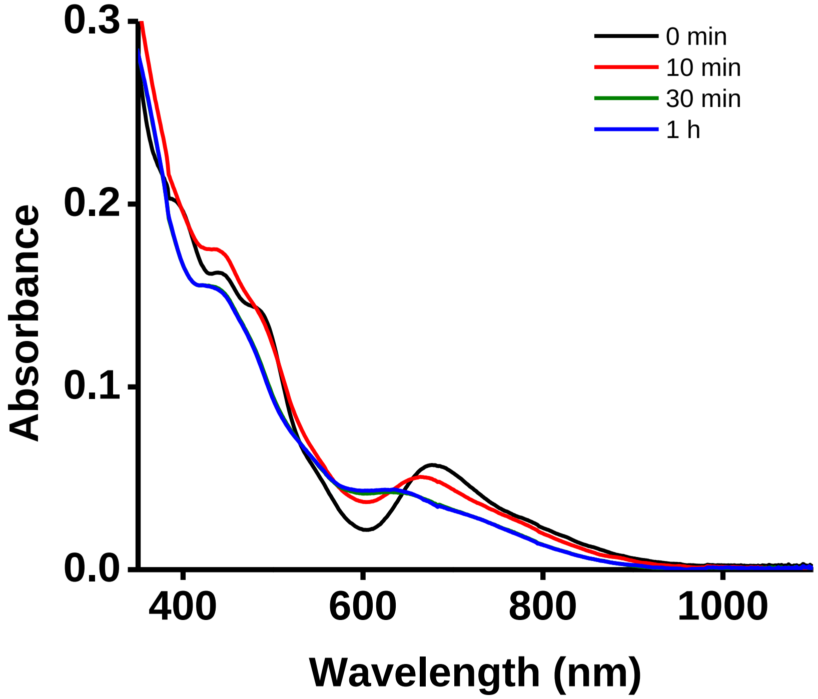

Supplementary Figure 15. Time-dependent change in the UV/Vis absorption spectra of a mixture of $Ag_{25}(DMBT)_{18}$ and $Au_{25}(PET)_{18}$at a molar ratio (Ag_{25}:Au_{25}) of 0.3:1.0. PET is 2-phenylethanethiol and DMBT is 2,4-dimethylbenzenethiol.

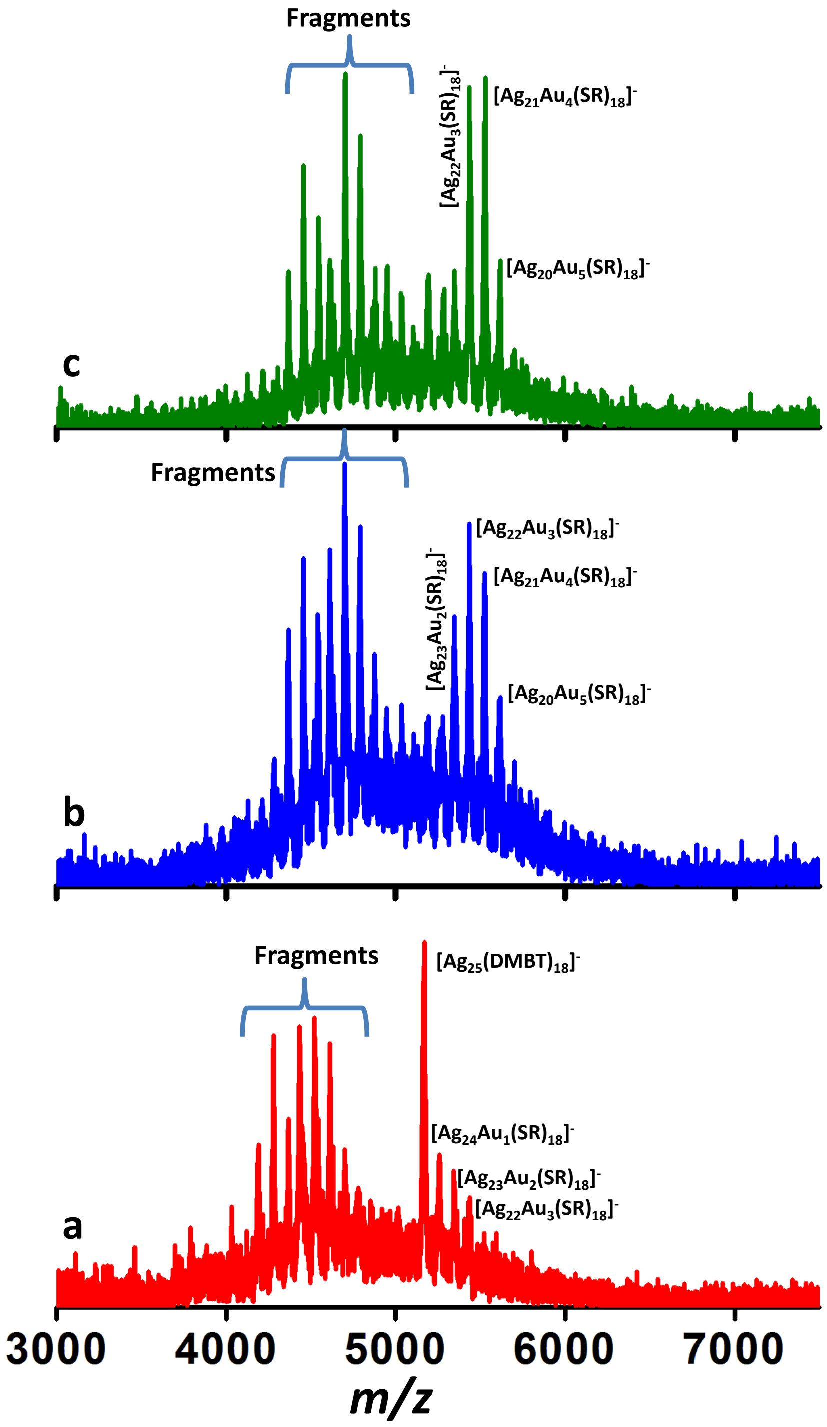

Supplementary Figure 16. Matrix assisted laser desorption ionization mass spectrometric (MALDI MS) analyses of 6.6:1.0 (Ag_{25}:Au_{25}) reaction mixture MALDI MS spectra of the reaction mixture of $Ag_{25}(DMBT)_{18}$ and $Au_{25}(PET)_{18}$ at molar ratio (Ag_{25}:Au_{25}) of 6.6:1.0

measured within 2 min after mixing (a) and after 10 min (b) and after 1 h (c). Since the mass of DMBT (2,4-dimethylbenzenethiol) and PET (2-phenylethanethiol) ligands are the same, exchange of these ligands between the clusters is not evident in the mass spectrum and hence the exact numbers of each of these ligands are unknown and hence SR is used instead of DMBT and PET in the peak labels. Spectra were measured at threshold laser intensity, however, fragmentation was observed.

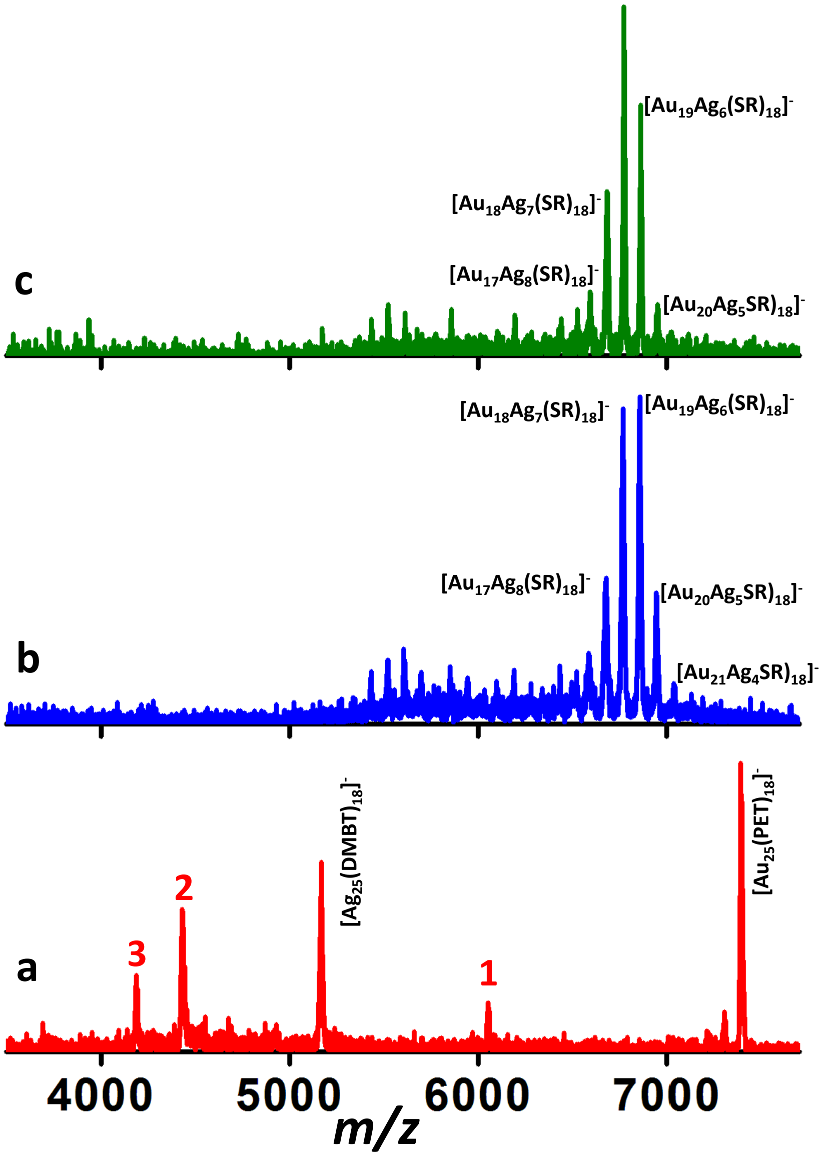

Supplementary Figure 17. Matrix assisted laser desorption ionization mass spectrometric (MALDI MS) analysis of 0.3:1.0 (Ag_{25}:Au_{25}) reaction mixture MALDI MS spectra of the

reaction mixture of $Ag_{25}(DMBT)_{18}$ and $Au_{25}(PET)_{18}$ at molar ratio (Ag_{25}:Au_{25}) of 0.3:1.0 measured within 2 min after mixing (a), after 10 min (b) and after 1 h (c). Since the mass of DMBT (2,4-dimethylbenzenethiol) and PET (2-phenylethanethiol) ligands are the same, exchange of these ligands between the clusters is not evident in the mass spectrum and hence the exact numbers of each of these ligands are unknown. Therefore, SR is used instead of DMBT and PET in the peak labels. The peak labeled as 1 in panel a is due to the fragment, $Au_{21}(PET)_{14}$, from $Au_{25}(PET)_{18}$, as shown previously in Supplementary Figure 3b. The peaks labeled as 2 and 3 are due to the fragments, $Ag_{22}(DMBT)_{15}$ and $Ag_{21}(DMBT)_{14}$, respectively, from $Ag_{25}(DMBT)_{18}$, as shown in Supplementary Figure 3a. As can be seen, reactions can be observed within 2 min making alloy clusters. Panels b and c show the same product features which indicate that the reaction was completed within about 10 min.

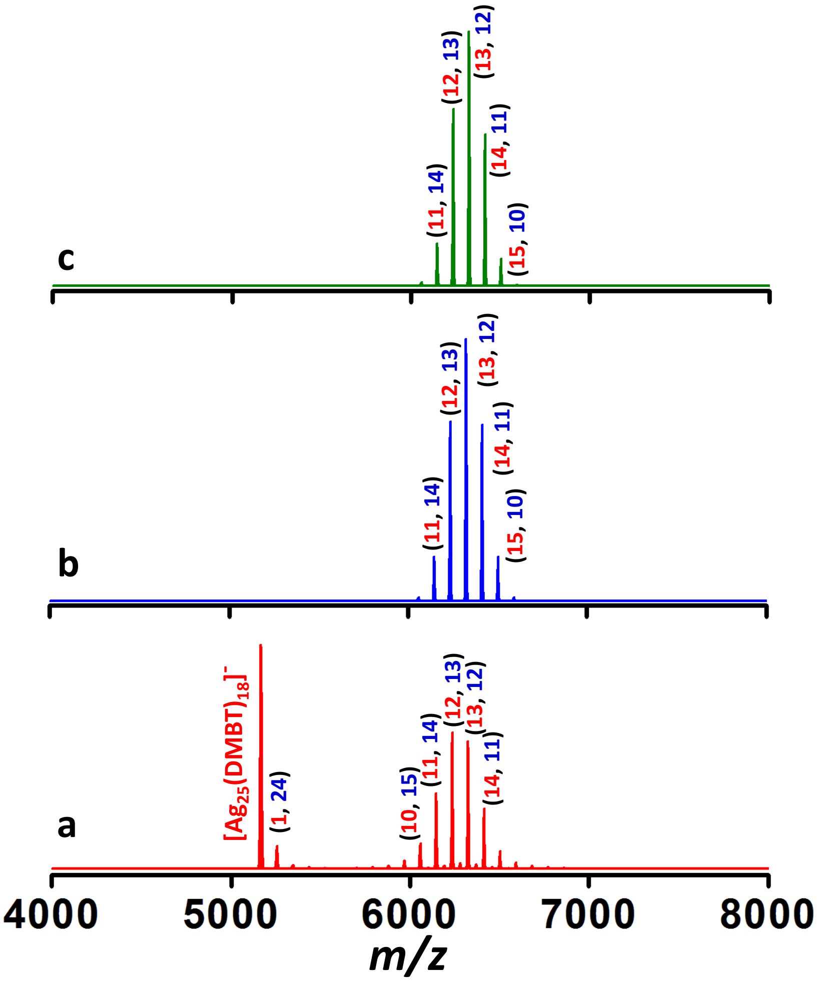

Supplementary Figure 18. Evolution of alloy clusters with intermediate level of doping. Time-dependent change in the electrospray ionization mass spectra (ESI MS) during the reaction between $Ag_{25}(DMBT)_{18}$ and $Au_{25}(PET)_{18}$ at a molar ratio (Ag_{25}:Au_{25}) of 1.0:1.0 within 2 min after mixing (a), after 10 min (b) and after 1 h (c). The peak labels given as numbers in red (*m*) and blue (*n*) in parentheses give the number of Ag and Au atoms, respectively in the alloy clusters, $Ag_mAu_n(SR)_{18}$. PET is 2-phenylethanethiol and DMBT is 2,4-dimethylbenzenethiol.

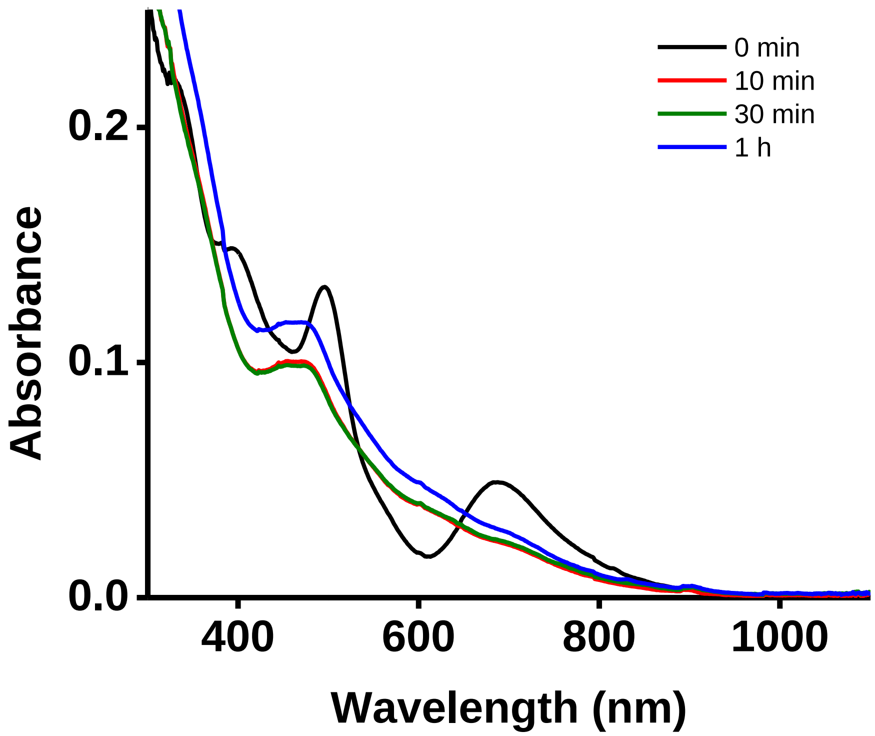

Supplementary Figure 19. Time-dependent change in the UV/Vis absorption spectra during the reaction between $Ag_{25}(DMBT)_{18}$ and $Au_{25}(PET)_{18}$ at a molar ratio (Ag_{25}:Au_{25}) of 1.0:1.0. PET is 2-phenylethanethiol and DMBT is 2,4-dimethylbenzenethiol.

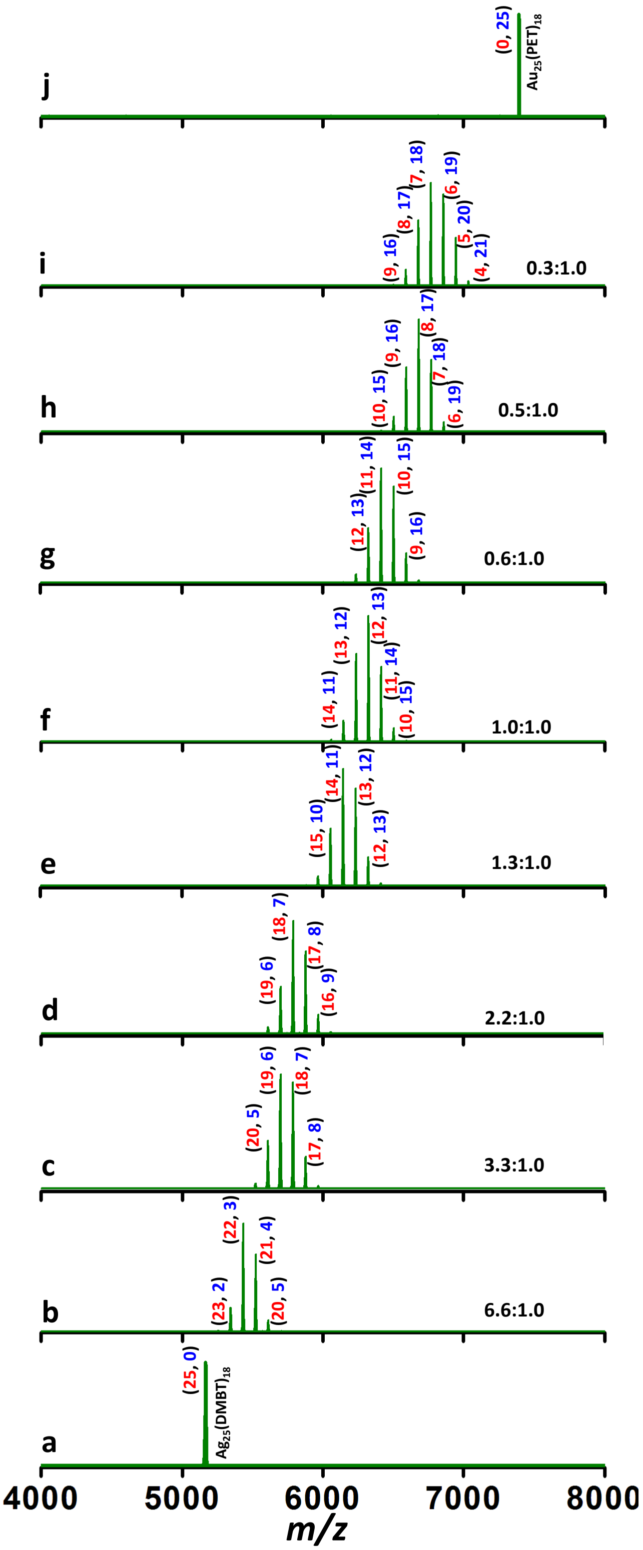
j
(0, 25)
$Au_{25}(PET)_{18}$
i
(9, 16)
(8, 17)
(7, 18)
(6, 19)
(5, 20)
(4, 21)
0.3:1.0
h
(10, 15)
(9, 16)
(8, 17)
(7, 18)
(6, 19)
0.5:1.0
g
(12, 13)
(11, 14)
(10, 15)
(9, 16)
0.6:1.0
f
(14, 11)
(13, 12)
(12, 13)
(11, 14)
(10, 15)
1.0:1.0
e
(15, 10)
(14, 11)
(13, 12)
(12, 13)
1.3:1.0
d
(19, 6)
(18, 7)
(17, 8)
(16, 9)
2.2:1.0
c
(20, 5)
(19, 6)
(18, 7)
(17, 8)
3.3:1.0
b
(23, 2)
(22, 3)
(21, 4)
(20, 5)
6.6:1.0
a
(25, 0)
$Ag_{25}(DMBT)_{18}$
4000
5000
6000
7000
8000
m/z

Supplementary Figure 20. Tuning the composition of $Ag_mAu_n(SR)_{18}$ clusters Electrospray ionization mass spectra (ESI MS) showing the equilibrium distribution of alloy clusters, $Ag_mAu_n(SR)_{18}$ formed at various Ag_{25}:Au_{25} molar ratios, as indicated in panels b-i. Panels a and j correspond to pure reactant clusters, $Ag_{25}(DMBT)_{18}$ and $Au_{25}(PET)_{18}$, respectively. PET is 2-phenylethanethiol and DMBT is 2,4-dimethylbenzenethiol. The spectra shown in panels b-i were measured about 1 h after (because product distribution does not change thereafter indicating the completion or equilibration of the reaction) mixing of the two reactant solutions. The peak labels given as numbers in red (*m*) and blue (*n*) in parentheses give the number of Ag and Au atoms, respectively, in the alloy clusters, $Ag_mAu_n(SR)_{18}$. The data show that $Ag_{25}(SR)_{18}$ can be transformed to $Ag_mAu_n(SR)_{18}$ clusters in the entire range, *n*=1-24.

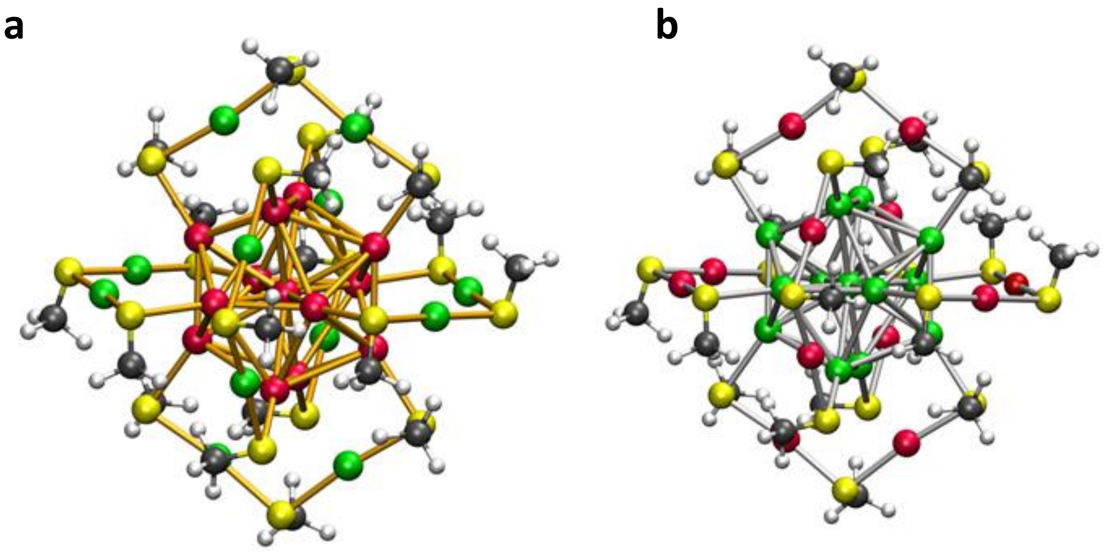

Supplementary Figure 21. Structures of $Au_{13}Ag_{12}(SR)_{18}$ and $Ag_{13}Au_{12}(SR)_{18}$ Schematic of the structures of $Au_{13}Ag_{12}(SR)_{18}$ (a) and $Ag_{13}Au_{12}(SR)_{18}$ (b). Note that the overall structures of these two clusters are the same, irrespective of the positions of the atoms. This also indicate that overall structures of the $Ag_mAu_n(SR)_{18}$ (m+n=25) clusters will be the same and irrespective of the values of *m* and *n*. Color codes of atoms: Red (Gold), Green (Silver), Yellow, (Sulfur), Black (Carbon) and White (Hydrogen). The –R group is taken as CH_3.

Supplementary Tables

Supplementary Table 1: Charges on the atoms of $[Ag_{25}(DMBT)_{18}]^-$. For the labels of atoms, please refer to Supplementary Figure 7. DMBT is 2,4-dimethylbenzenethiol.

**Atom**	**Corrected partial charges**
Ag (center)	+0.038000
Ag (icosahedron)	0.106465
Ag (staples)	0.325465
CA1	0.004031
CA6	-0.167510
CA2	0.024025
CA5	-0.169990
CA3	-0.256960
CT1	-0.082780
CA4	0.010246
CT2	-0.097200
HA3	0.147461
HA1	0.155527
HT3	0.035745
HT1	0.036475
HT2	0.034854
HA2	0.131897
HT6	0.052728
HT5	0.049215
HT4	0.035223

S	-0.288600

Supplementary Table 2: Charges on the atoms of $[Au_{25}(PET)_{18}]^{-}$. For the labels of atoms, please refer to Supplementary Figure 7. PET is 2-phenylethanethiol.

Atom	Corrected partial charge
Au (center)	-0.08
Au (icosahedron)	0.038729
Au (staples)	0.124729
CT1	-0.027855
HT1	0.039546
HT2	0.136658
CT2	0.0150473
HT3	0.037078
HT4	0.026858
CA1	-0.004147
CA2	-0.133917
HA2	0.146177
CA4	-0.173749
HA4	0.143557
CA6	-0.101539
HA6	0.131833
CA5	-0.149795
HA5	0.141473
CA3	-0.186006
HA3	0.160190
S	-0.361495

Supplementary Table 3: Comparison of average bond distances between isolated clusters and the adduct PET is 2-phenylethanethiol and DMBT is 2,4-dimethylbenzenethiol. See Supplementary Fig. 13 and Supplementary Ref. 1 below in main manuscript for the description of longer and shorter M-M bond in icosahedral surface of $Au_{25}(SR)_{18}$ and $Ag_{25}(SR)_{18}$. The bond distances for the crystal structures of $Au_{25}(PET)_{18}$ and $Ag_{25}(DMBT)_{18}$ were taken from the Supplementary Ref. 2 and 3, respectively.

	**All M-S bonds (M=Ag/Au) (Å)**	**M-S bonds in staples only (Å)**	**$M_{icosahedral}$-S bonds (Å)**	**Shorter M-M bonds on the I positions (M=Ag/Au) (Å)**	**Longer M-M bonds on the I positions (M=Ag/Au) (Å)**	**Central M-icosahedral M distances (Å)**
**$Au_{25}(PET)_{18}$ From crystal structure**		2.32±0.01	2.38±0.01			2.79±0.01
**$Au_{25}(PET)_{18}$ DFT-optimized**	2.39	2.36	2.44	2.82	3.05	2.85
**$Au_{25}(PET)_{18}$ in DFT-optimized adduct**	2.49	2.46	2.56	2.82	3.09	2.89
**$Ag_{25}(DMBT)_{18}$ Crystal structure**		2.445─2.946	2.453─2.510			2.7486─2.7847
**$Ag_{25}(DMBT)_{18}$ DFT-optimized**	2.48	2.45	2.56	2.90	3.00	2.83
**$Ag_{25}(DMBT)_{18}$ in DFT-optimized adduct**	2.56	2.53	2.62	3.03	3.05	2.90

Supplementary Table 4: DFT energies of undoped clusters and isolated metal atoms. Values for Ag and Au are taken from Supplementary Ref. 4 below. E_0 is the DFT-optimized energies of the individual clusters and isolated Ag/Au atoms. PET is 2-phenylethanethiol and DMBT is 2,4-dimethylbenzenethiol.

**Structure**	E_0**/eV**
$Ag_{25}(DMBT)_{18}$	-2033.613
$Au_{25}(PET)_{18}$	-2045.064
Ag	-0.186
Au	-0.203

Supplementary Table 5: Isomers of $Ag_{24}Au(DMBT)_{18}$ and their DFT energies. E is the DFT-optimized energies of indivdual isomers (C, I and S) of $Ag_{24}Au(DMBT)_{18}$. ΔE is the difference between the energy of $Ag_{24}Au(DMBT)_{18}$ and that of $Ag_{25}(DMBT)_{18}$. DMBT is 2,4-dimethylbenzenethiol.

**Isomer**	**Location of Au in $Ag_{24}Au(DMBT)_{18}$**	**Energy E/eV**	**ΔE/meV**
C	Centre of icosahedron	-2034.535	-921.4
I	Icosahedron	-2034.171	-557.3
S	Staple	-2034.209	-595.5

Supplementary Table 6: Isomers of $Au_{24}Ag(PET)_{18}$ and their DFT energies. E is the DFT-optimized energies of indivdual isomers (C, I and S) of alloy clusters. ΔE is the difference between the energy of $Au_{24}Ag(PET)_{18}$ and that of $Au_{25}(PET)_{18}$. PET is 2-phenylethanethiol.

**Isomer**	**Location of Ag in $Au_{24}Ag(PET)_{18}$**	**Energy E/eV**	**ΔE/meV**
C	Centre of icosahedron	-2044.651	+413.7
I	Icosahedron	-2045.091	-27.1
S	Staple	-2044.822	+241.5

Supplementary Table 7: DFT energies of the substitution reaction, Au + $Ag_{25}(DMBT)_{18}$→ $Ag_{24}Au(DMBT)_{18}$ + Ag. ΔE_s is the energy of the corresponding substitution reaction mentioned above. PET is 2-phenylethanethiol and DMBT is 2,4-dimethylbenzenethiol.

**Isomer**	**Position of Au in $Ag_{24}Au(DMBT)_{18}$**	**ΔE_s/meV**
C	Centre of icosahedron	-904.4
I	Icosahedron	-540.3
S	Staples	-578.5

Supplementary Table 8: DFT energies of the substitution reaction, Ag +$Au_{25}(PET)_{18}$ → $Au_{24}Ag(PET)_{18}$ + Au. ΔE_s is the energy of the corresponding substitution reaction mentioned above. PET is 2-phenylethanethiol and DMBT is 2,4-dimethylbenzenethiol.

**Isomer**	**Position of Ag in $Au_{24}Ag(PET)_{18}$**	**ΔE_s/meV**
C	Centre of icosahedron	+396.7
I	Icosahedron	-44.1
S	Staples	+224.5

Supplementary Notes

Supplementary Note 1: Possible reason for the abundance of $Ag_{13}Au_{12}(SR)_{18}$

Our DFT calculations show that substitution energy for an Au atom to occupy the I and the S positions of $Ag_{25}(SR)_{18}$ are almost the same which indicates that Au_{12} can be located in positions I or the S of $Ag_{25}(SR)_{18}$ with equal probability. Thus substitution of the twelve staple (S)/ icosahedral (I) Ag atoms in $Ag_{25}(SR)_{18}$ by twelve Au atoms produce $Ag_{13}Au_{12}(SR)_{18}$. Hence, more of $Ag_{13}Au_{12}(SR)_{18}$ could be formed as a result of Au substitution into $Ag_{25}(SR)_{18}$ on either at the I or at the S positions. Therefore, the probability of formation of $Ag_{13}Au_{12}(SR)_{18}$ is higher due to the availability of two types of (I and S) twelve-atom sites for Au atoms.

Further, $Ag_{13}Au_{12}(SR)_{18}$ can also be derived from $Au_{25}(SR)_{18}$ as a result of Ag substitution. Our DFT calculations shows that an Ag atom prefers to occupy the I position rather than the S positions. The complete substitution of all the I positions in $Au_{25}(SR)_{18}$ by Ag atoms would result in the formation of $Ag_{12}Au_{13}(SR)_{18}$. The thirteenth Ag atom can occupy any one of the twelve S positions in the staples (note that C site (center of icosahedron) is least preferred for Ag atom substitution), resulting in the formation of $Ag_{13}Au_{12}(SR)_{18}$. Thus the probability of the formation of $Ag_{13}Au_{12}(SR)_{18}$ is higher due to the availability of the twelve staple (S) sites for the thirteenth Ag atom.

We do not think that the abundance of $Ag_{13}Au_{12}(SR)_{18}$ is due to any shell closing effects as this abundance is observed only when the concentrations of the reacting clusters are comparable. Though this species was observed with higher abundance immediately after mixing (Figure 1c), and it existed for about 5 min (Supplementary Figure 6), no such species was observed after 1h (panel i of Supplementary Figure 20). Further, Supplementary Figure 20 shows that $Ag_{13}Au_{12}$ was not observed with any significantly higher abundance (even at higher concentrations of Au_{25}), in contrast to what is seen in Figure 1c and Supplementary Figure 6. These observations show that significantly higher abundance of $Ag_{13}Au_{12}$ is observed only for a few minutes after mixing the clusters. As the reaction proceeds, this species also undergoes further doping. If the observed abundance of $Ag_{13}Au_{12}(SR)_{18}$ is due to its higher stability due to any shell closing effects, this species is expected to remain at higher abundance for longer time intervals of the reaction.

In summary, the $Ag_{13}Au_{12}(SR)_{18}$ detected can be due to a number of isomers depending on (i) the cluster from which it is derived and (ii) the exact locations of the Ag_{12}/Au_{12} and the thirteenth Ag/Au atom. However, standard mass spectrometry cannot distinguish all the isomers of the formula, $Ag_{13}Au_{12}(SR)_{18}$. We think that the abundance of $Ag_{13}Au_{12}(SR)_{18}$ could be due to the larger number of ways by which $Ag_{13}Au_{12}(SR)_{18}$ can be formed.

Supplementary Note 2: General instrumental parameters used for ESI measurements

All samples were analyzed by Waters Synapt G2Si High Definition Mass Spectrometer equipped with electrospray ionization (ESI) and ion mobility (IM) separation. All the samples were analyzed in negative ESI mode. The instrumental parameters were first optimized for $Au_{25}(PET)_{18}$ and other samples were analyzed using the similar setting with slight modification depending on the sample. About 0.1 mg of as prepared samples were diluted with dichloromethane (DCM) to get about 10 µg/mL sample concentration and directly infused to the system without any further purification. The instrument was calibrated using NaI as calibrant for the high mass. The optimized conditions were as follows:

Sample concentration: 10 µg /mL
Diluents: DCM
Sample flow rate: 10-20 µL/min
Source Voltage: 1.5-2.5 kV
Cone Voltage: 120-140 V
Source Offset: 80-120 V
Trap Collision Energy: 0 V
Transfer Collision Energy: 0 V
Source Temperature: 80-100°C
Desolvation Temperature: 150-200°C
Desolvation Gas Flow: 400 L/h
Trap Gas Flow: 2mL/min

Supplementary Note 3: Instrumental parameters used for the detection of the $Ag_{25}Au_{25}(DMBT)_{18}(PET)_{18}$ adduct.

By slightly modifying the instrumental setting, we were able to identify $[Ag_{25}Au_{25}(DMBT)_{18}(PET)_{18}]^{2-}$. The conditions are as follows:

Sample concentration: 10 µg /mL
Diluents: DCM

Sample flow rate: 10-20 µL/min

Source Voltage: 0.1-0.5 kV

Cone Voltage: 0-50 V

Source Offset: 0-20 V

Trap Collision Energy: 0 V

Transfer Collision Energy: 0 V

Source Temperature: 60-80°C

Desolvation Temperature: 100-150°C

Trap Gas Flow: 5 mL/min

Supplementary Note 4: Methods of molecular docking study of $Au_{25}(PET)_{18}$ and $Ag_{25}(DMBT)_{18}$

We used the Autodock 4.2 and AutoDock Tools programs[5]. The initial structures of the molecules were the crystal structures of both $Ag_{25}(DMBT)_{18}$ and $Au_{25}(PET)_{18}$[6, 2]. $Au_{25}(PET)_{18}$ was taken as the “ligand” i.e. the movable molecule whose degrees of freedom would be varied and $Ag_{25}(DMBT)_{18}$ as the “receptor” which was the fixed and completely rigid central molecule. We assigned charges by following the procedure to Guberman-Pfeffer *et al.*[7], with slight modifications. The charges of the C, H and S atoms of the ligands were derived from a two-stage RESP fitting procedure based on partial charges and geometry optimization of the protonated PET and DMBT ligands at B3LYP/6-31G(d,p) level of theory. We used the Bader partial charges of Au and Ag atoms from optimized structures of $Au_{25}(SCH_3)_{18}$ and $Ag_{25}(SCH_3)_{18}$. The optimization was carried out using GPAW with the PBE functional[8] and DZP (double zeta plus polarization) LCAO basis set. The $Ag(4d^{10}5s^1)$, $Au(5d^{10}6s^1)$, and $S(3s^23p^4)$ electrons were treated as valence and the inner electrons were included in a frozen core. The GPAW setups for gold and silver included scalar-relativistic corrections. The grid spacing for the finite difference method was 0.2 Å. The convergence criterion of 0.05 eV/Å for the residual forces on atoms was used for the structure optimization, without any symmetry constraints. After optimization using an LCAO basis set, the finite difference grid method of GPAW was used to obtain more accurate values of electronic density for Bader charge calculations. The same method was applied to $Ag_{25}(DMBT)_{18}$ also. We made a correction to the calculated charges following Guberman-Pfeffer et al.[7], except that we applied the charge correction factor for all the atoms except the central atom and hydrogen atoms. The equation used for obtaining the correction factor (δq) was:

$Q_{\text{total}} = 25Q_{(Au/Ag)} + 144Q_{C} + 18Q_{S} + 162Q_{H} + 186\delta q$……..(1)

where, Q_{total}= total charge on the cluster (-1) and $Q_{(Au/Ag)}$, Q_C, Q_S, Q_H are the charge on metal atoms, carbon, sulfur and hydrogen atoms obtained by Bader method.

Hence, $\delta q = \{Q_{total} - [25Q_{Au/Ag} + 144Q_{C} + 18Q_{S} + 162Q_{H}]\}/186$. The division by 186 is because we have not applied the correction factor to the Bader charges on the metal atom in the center of icosahedra and the hydrogen atoms on the ligands. The correction factors obtained were 0.000373*e* for $Au_{25}(PET)_{18}$ and 0.020535*e* for $Ag_{25}(DMBT)_{18}$.

The van der Waals radius σ (Å) and well depth ε (kcal/mol) for Ag and Au of 2.63 Å and 2.96 Å and 4.560 kcal/mol and 5.295 kcal/mol, respectively, were taken from well-tested sources in literature[9,10] and these atom types were added to the Autodock parameter file which does not contain them by default.

Since the computation of many torsional configurations of the ligands using a force-field increases the configuration space and therefore the number of computations required greatly, the use of molecular docking software is highly advantageous as it is specifically designed for speeding up the process of calculating torsions by the use of pre-calculated grid maps of the force-field of the receptor and ligand. The reason for choosing $Au_{25}(PET)_{18}$ as the movable and flexible molecule was that PET ligands have a larger torsional flexibility than the DMBT ligands, which would result in lower energy minima during the optimization over the torsional degrees of freedom.

The size of the search space in which $Au_{25}(PET)_{18}$ was to be moved was a cube with a side of length 126 points with point spacing 0.375 Å. The cube was centered on the central silver atom of $Ag_{25}(DMBT)_{18}$. The initial configuration of the clusters was chosen in a random orientation and positioned a sufficient distance away from the parent cluster so that binding interactions would be minimal. The Lamarckian genetic algorithm method was used within Autodock to find the global minimum with respect to the positional, orientation and torsional degrees of freedom of $Au_{25}(PET)_{18}$ keeping $Ag_{25}(DMBT)_{18}$ fixed at the center of the box. We have treated all the M-M, M-S, C-C, and C-H bonds as rigid, while allowing torsional flexibility upto 54 torsions on the S-C1, C1-C2 and C2-C3 bonds on all 18 of the ligands of the $Au_{25}(PET)_{18}$ cluster, where the carbons are numbered along the chain starting from the S end (See Supplementary Fig. 8).

The 10 lowest minima structures that were obtained with binding free energies ranging from -4.62 to -17.53 kcal/mol with $Au_{25}(PET)_{18}$ in various orientations and at varying distances on various different sides the $Ag_{25}(DMBT)_{18}$. The free energies of binding were calculated by summing the intermolecular and internal and torsional terms and subtracting the unbound energy which is a calculation that is performed within the Autodock program. The relative orientation of the clusters in the minimum energy configuration is shown in Supplementary Fig. 11 and is such that the overlap between the molecular surfaces, or van der Waals envelope, of the ligands of the two clusters has maximal area of contact and their protrusions and pocket fit closely together in a lock and key fashion, as can be seen in Supplementary Fig 10. Two of the C_5 axes are nearly aligned with each other as seen in Supplementary Fig 11. Strength of interactions between atoms in the staples of the two clusters in the minimum energy configuration can be partially gauged by comparing their interatomic distances to the sum of their van der Waals radii, since the attractive van der Waals interaction ($-A/r^{-6}$) is stronger than the repulsive term (B/r^{-12}) at distances close to and greater their van der Waals radii.

Supplementary Note 5: Justification for not including the van der Waals corrections in the DFT-optimization of the force-field global minimum geometry of $[Ag_{25}Au_{25}(DMBT)_{18}(PET)_{18}]^{2-}$

Since the orientations of the ligands in the Autodock force-field minimum of $[Ag_{25}Au_{25}(DMBT)_{18}(PET)_{18}]^{2-}$ (see Supplementary Fig. 9), in which the vdW potential is explicitly included, are very similar to the orientation of the ligands in the DFT-optimized (with no vdW corrections) structure (see Fig. 3 in main manuscript), we concluded that the addition of vdW corrections to DFT optimization will not make a significant difference to the structure of the ligands of the dimer, or in other parts of the structure. Besides, our focus is not the dimer intermediate but the reaction leading to metal exchange and that finding does not get affected by improving the accuracy of the dimer structure by including the van der Waals corrections in the DFT optimization.

Supplementary Note 6: Nature of the bonding in the adduct $[Ag_{25}Au_{25}(DMBT)_{18}(PET)_{18}]^{2-}$

The shortest distances between the pairs of different types of metal and sulfur atoms (S-Au, S-Ag, Au-Ag) in the staples, shown in Supplementary Fig. 9, of the two clusters in the adduct

show that all these interatomic distances are greater than those needed for covalent bonding. Thus in our force-field minimum geometry, only weak non-covalent interactions between (i) metal atoms of one cluster and sulfur atoms in the staples of the neighbouring cluster and (ii) the alkyl/aryl groups of the ligands of the clusters are only expected in this force-field global minimum geometry of adduct at this separation between **I** and **II**, unlike in the case of one-dimensional chains of clusters[11].

However, DFT optimization of the force-field global minimum of adduct (Supplementary Fig. 12 and 13) revealed that the clusters **I** and **II** undergo significant structural distortions in the adduct geometry in terms of the bond lengths and bond angles. Moreover, the DFT-optimized geometry shows the formation of a weak bonding between Ag atom and the sulfur atoms in the staples of **I** and **II**. However, the local minimum in DFT-PES, shown in these figures, cannot be confirmed as the actual global minimum of the DFT PES of the dimer, without a complete search of PES but it is reasonable to assume that this would at least resemble in essential aspects such as the overall separation and orientation of the two clusters to one of the lower lying minima in the DFT PES.

The energy difference (binding energy) between the DFT-optimized adduct geometry and the sum of the DFT-optimized energies of isolated clusters was -90 eV. A quite significant lowering of the total energy of the cluster is observed. However, the per atom binding energy is 90/698 eV = 0.12 eV (698 is the total number of atoms in the adduct, $[Ag_{25}Au_{25}(DMBT)_{18}(PET)_{18}]^{2-}$). The large magnitude of the binding energy can be attributed to a number of factors such as the presence of large number of atoms and large common interfacial area, which facilitates attractive binding interactions, such Ag-S(Au_{25}) chemical bonding in the adduct, pi-pi interactions, ligand orbital overlaps, dipole-dipole interactions. Secondly, there is additional structural relaxation taking place through the structures resulting in distortions such as bond strain, angular and dihedral distortions in the core and staples, which lower the total energy below that of the sum of the DFT-optimized geometry of the two isolated clusters. However, we are unable to decompose the total energy into these different contributions.
Such a substantial energy reduction for an intermediate indicates that the overall reaction is also thermodynamically favorable.

Supplementary Note 7: MALDI MS measurements

The matrix used was trans-2-[3-(4-tertbutylphenyl)-2-methyl-2-propenylidene]malononitrile (DCTB, > 98%). A solution of 6.2 mg of DCTB in 0.5 mL of dicholoromethane was used for the measurements. Appropriate volumes of the sample solutions and DCTB solutions in DCM were mixed thorougly and spotted on the sample plate and allowed to dry at ambient conditions. All the MALDI MS measurements were carried out at the threshold laser fluence in order to minimize fragmentation. All the spectra reported are of negative ions.

Supplementary References:

1. Yamazoe, S. *et al.* Hierarchy of bond stiffnesses within icosahedral-based gold clusters protected by thiolates. *Nat. Commun.* **7,** 10414 (2016).

2. Heaven, M. W., Dass, A., White, P. S., Holt, K. M. & Murray, R. W. Crystal structure of the gold nanoparticle $[N(C_8H_{17})_4][Au_{25}(SCH_2CH_2Ph)_{18}]$. *J. Am. Chem. Soc.* **130,** 3754-3755 (2008).

3. Bootharaju, M. S., Joshi, C. P., Parida, M. R., Mohammed, O. F. & Bakr, O. M. Templated atom-precise galvanic synthesis and structure elucidation of a $[Ag_{24}Au(SR)_{18}]^-$ nanocluster. *Angew. Chem. Int. Ed.* **55,** 922-926 (2016).

4. Krishnadas, K. R., Ghosh, A., Baksi, A., Chakraborty, I., Natarajan, G. & Pradeep, T. Intercluster reactions between $Au_{25}(SR)_{18}$ and $Ag_{44}(SR)_{30}$. *J. Am. Chem. Soc.* **138,** 140-148 (2016).

5. Morris, G. M., Huey, R., Lindstrom, W., Sanner, M. F., Belew, R. K., Goodsell, D. S. & Olson, A. J. Autodock4 and AutoDockTools4: automated docking with selective receptor flexibility. *J. Comput. Chem.* **16,** 2785-2791 (2009).

6. Joshi, C. P., Bootharaju, M. S., Alhilaly, M. J. & Bakr, O. M. $[Ag_{25}(SR)_{18}]^-$: The "Golden" silver nanoparticle**.** *J. Am. Chem. Soc.* **137,** 11578-11581 (2015).

7. Guberman-Pfeffer, M. J., Ulcickas, J. & Gascón, J. A. Connectivity-based biocompatible force field for thiolated gold nanoclusters *J. Phys. Chem. C* **119,** 27804−27812 (2015).

8. Perdew, J. P., Burke, K. & Ernzerhof, M. Generalized gradient approximation made simple. *Phys. Rev. Lett.* **77,** 3865-3868 (1996).

9. Pohjolainen, E., Chen, X., Malola, S., Groenhof, G. & Häkkinen, H. A unified AMBER-compatible molecular mechanics force field for thiolate-protected gold nanoclusters *J. Chem. Theory Comput.* **12,** 1342−1350 (2016).

10. Heinz, H., Vaia, R. A., Farmer, B. L. & R. R. Naik Accurate simulation of surfaces and interfaces of face-centered cubic metals using 12−6 and 9−6 Lennard-Jones potentials. *J. Phys. Chem. C* **112,** 17281–17290 (2008).

11. Nardi, M. D. *et al.* Gold nanowired: a linear $(Au_{25})_n$ polymer from Au_{25} molecular clusters. *ACS Nano* **8,** 8505-8512 (2014).
