# Supplementary material for: Structure-conserving spontaneous transformations between nanoparticles
Source: Nat Commun. 2016 Nov 10;7:13447. doi: 10.1038/ncomms13447 (PMC5110647; doi:10.1038/ncomms13447)
Supplement: Supplementary Data 1 — Coordinates of the force-field global minimum energy configuration of the adduct [Ag25Au25(DMBT)18(PET)18]2-. [file ncomms13447-s2.docx]

**Coordinates of the force-field global minimum energy configuration of the adduct** [Ag_25_Au_25_(DMBT)_18_(PET)_18_]^2-^.

698

Au 18.958 3.766 0.310

Au 16.870 6.283 0.275

Au 13.923 4.881 -1.358

Au 15.167 1.993 -2.398

Au 19.916 3.127 -2.571

Au 22.079 3.996 -0.102

Au 20.417 2.803 2.928

Au 17.838 1.208 1.533

Au 17.135 4.317 -2.117

Au 18.971 5.018 -4.820

Au 20.759 1.862 -5.365

Au 19.668 0.136 -2.823

Au 16.664 -0.454 -2.900

Au 18.763 -2.953 -2.940

Au 21.725 -1.560 -1.337

Au 20.378 1.261 -0.118

Au 15.742 0.080 -0.100

Au 13.557 -0.625 -2.438

Au 15.260 0.562 -5.532

Au 17.705 2.151 -4.091

Au 18.454 -0.985 -0.445

Au 16.652 -1.714 2.214

Au 14.860 1.430 2.713

Au 15.995 3.218 0.240

Au 17.801 1.668 -1.297

S 19.206 6.243 0.591

S 14.545 6.655 0.070

S 13.120 3.285 -2.909

S 22.321 4.012 2.237

S 18.647 1.555 3.889

S 22.116 4.263 -2.451

S 17.129 6.136 -3.818

S 20.749 4.113 -6.110

S 21.027 -0.428 -4.871

S 16.422 -2.912 -3.230

S 21.093 -3.339 -2.761

S 22.539 0.069 0.196

S 13.368 -0.658 -4.796

S 17.053 1.805 -6.479

S 13.453 -0.900 -0.071

S 18.483 -2.832 1.212

S 14.885 -0.804 3.500

S 14.539 3.707 2.211

C 19.460 6.569 2.398

C 20.111 7.932 2.665

C 21.586 8.023 2.355

C 22.072 7.800 1.058

C 23.441 7.866 0.795

C 24.343 8.171 1.817

C 23.870 8.402 3.109

C 22.502 8.315 3.371

C 22.227 -0.827 1.799

C 23.316 -1.864 2.097

C 23.755 -1.867 3.541

C 24.778 -2.723 3.983

C 25.163 -2.739 5.325

C 24.534 -1.900 6.248

C 23.516 -1.045 5.822

C 23.130 -1.034 4.480

C 17.786 -4.314 0.348

C 17.131 -5.297 1.328

C 18.145 -5.828 2.312

C 18.728 -7.090 2.115

C 19.604 -7.629 3.058

C 19.907 -6.917 4.220

C 19.335 -5.660 4.424

C 18.465 -5.118 3.477

C 13.725 -2.742 -0.010

C 12.384 -3.430 0.277

C 12.521 -4.738 1.013

C 11.425 -5.589 1.214

C 11.573 -6.797 1.897

C 12.823 -7.176 2.389

C 13.920 -6.331 2.213

C 13.765 -5.122 1.534

C 15.843 4.560 3.219

C 15.222 5.326 4.387

C 16.146 6.373 4.956

C 16.298 7.620 4.331

C 17.102 8.611 4.901

C 17.776 8.367 6.100

C 17.642 7.126 6.724

C 16.829 6.141 6.158

C 19.473 -0.045 4.362

C 19.517 -0.262 5.882

C 18.577 -1.322 6.397

C 18.044 -1.284 7.694

C 17.206 -2.305 8.145

C 16.887 -3.376 7.310

C 17.410 -3.423 6.015

C 18.247 -2.401 5.567

C 16.185 -3.248 -5.036

C 14.738 -3.636 -5.356

C 14.468 -5.118 -5.301

C 14.990 -5.915 -4.274

C 14.736 -7.287 -4.231

C 13.974 -7.889 -5.234

C 13.457 -7.106 -6.268

C 13.695 -5.733 -6.296

C 14.503 8.108 -1.087

C 15.181 9.335 -0.474

C 15.350 10.408 -1.521

C 14.459 11.486 -1.616

C 14.662 12.503 -2.551

C 15.766 12.462 -3.406

C 16.658 11.392 -3.322

C 16.451 10.378 -2.386

C 21.111 -4.798 -1.609

C 22.538 -5.167 -1.192

C 22.540 -5.938 0.103

C 22.519 -7.340 0.087

C 22.555 -8.073 1.272

C 22.613 -7.414 2.501

C 22.638 -6.018 2.534

C 22.603 -5.290 1.344

C 19.729 -1.239 -5.914

C 20.323 -1.986 -7.110

C 19.809 -3.401 -7.196

C 18.440 -3.653 -7.377

C 17.958 -4.960 -7.501

C 18.846 -6.037 -7.456

C 20.212 -5.799 -7.276

C 20.689 -4.494 -7.144

C 17.821 7.614 -2.947

C 18.758 8.426 -3.852

C 19.621 9.359 -3.036

C 19.703 9.246 -1.640

C 20.428 10.170 -0.886

C 21.096 11.221 -1.519

C 21.033 11.336 -2.907

C 20.297 10.418 -3.659

C 13.419 4.172 -4.502

C 12.217 5.048 -4.875

C 12.620 6.341 -5.537

C 13.367 7.294 -4.829

C 13.727 8.505 -5.418

C 13.342 8.787 -6.729

C 12.598 7.847 -7.449

C 12.238 6.634 -6.858

C 15.770 -0.588 5.121

C 14.847 -0.663 6.345

C 13.489 -1.173 5.922

C 12.391 -0.305 5.828

C 11.165 -0.751 5.333

C 11.010 -2.079 4.931

C 12.090 -2.957 5.030

C 13.312 -2.506 5.527

C 12.012 0.547 -5.153

C 10.632 -0.067 -4.923

C 9.730 0.893 -4.191

C 8.557 0.423 -3.580

C 7.726 1.290 -2.873

C 8.051 2.644 -2.767

C 9.218 3.122 -3.368

C 10.053 2.250 -4.069

C 23.673 2.786 2.568

C 24.992 3.191 1.908

C 25.878 3.893 2.906

C 25.389 4.969 3.664

C 26.217 5.638 4.563

C 27.546 5.243 4.729

C 28.043 4.175 3.983

C 27.213 3.512 3.078

C 19.824 3.915 -7.706

C 20.383 4.832 -8.798

C 20.335 6.267 -8.339

C 21.299 6.778 -7.459

C 21.315 8.134 -7.127

C 20.360 8.999 -7.663

C 19.386 8.500 -8.532

C 19.376 7.146 -8.860

C 16.263 3.399 -7.031

C 15.571 3.318 -8.400

C 16.193 2.330 -9.352

C 17.558 2.389 -9.666

C 18.133 1.450 -10.523

C 17.353 0.437 -11.083

C 15.990 0.369 -10.780

C 15.418 1.312 -9.926

C 21.880 6.097 -2.510

C 23.138 6.739 -3.108

C 24.005 5.750 -3.844

C 25.231 5.318 -3.321

C 26.025 4.404 -4.016

C 25.604 3.909 -5.252

C 24.378 4.320 -5.780

C 23.587 5.232 -5.079

Ag 7.634 10.444 4.149

Ag 10.562 11.591 4.957

Ag 12.549 8.615 5.724

Ag 10.172 6.412 5.687

Ag 7.806 8.317 1.887

Ag 9.222 9.284 6.472

Ag 7.480 9.354 9.243

Ag 4.540 7.362 8.514

Ag 5.383 6.306 5.588

Ag 7.890 4.735 5.178

Ag 4.985 3.535 4.276

Ag 3.132 6.614 3.421

Ag 5.391 8.793 3.594

Ag 9.273 5.978 2.815

Ag 6.252 5.935 2.880

Ag 7.976 5.747 0.112

Ag 10.919 7.831 0.894

Ag 10.152 8.852 3.752

Ag 7.780 7.591 4.666

Ag 6.021 11.227 1.667

Ag 4.723 11.467 5.035

Ag 6.267 9.136 6.485

Ag 9.401 4.060 7.719

Ag 7.730 6.851 7.437

Ag 10.813 3.800 4.305

S 8.466 12.678 4.946

S 12.825 10.882 5.088

S 12.636 6.308 6.286

S 9.730 9.988 8.853

S 5.288 8.972 10.062

S 3.342 5.901 7.088

S 7.061 2.427 4.572

S 2.774 4.349 3.963

S 2.941 8.934 2.973

S 5.740 5.075 0.565

S 7.461 10.108 0.171

S 4.385 10.678 7.238

S 10.170 6.245 -0.675

S 12.165 9.352 2.237

S 7.842 5.053 9.202

S 4.761 12.695 3.014

S 11.144 4.302 1.999

S 10.757 2.663 6.383

C 6.915 1.321 5.974

C 8.019 0.502 6.253

C 5.742 1.172 6.724

C 7.952 -0.449 7.268

C 5.681 0.211 7.731

C 4.881 1.808 6.517

C 6.779 -0.604 8.010

C 6.722 -1.351 8.799

C 10.005 7.083 -2.253

C 10.489 8.377 -2.473

C 9.431 6.365 -3.310

C 10.386 8.949 -3.741

C 10.934 8.929 -1.645

C 9.336 6.943 -4.575

C 9.812 8.238 -4.796

C 9.732 8.696 -5.780

C 8.517 13.954 3.693

C 7.502 14.919 3.746

C 6.722 14.830 4.498

C 7.496 15.978 2.841

C 8.504 16.089 1.883

C 8.497 16.913 1.173

C 9.514 15.127 1.833

C 9.525 14.059 2.728

C 3.086 12.834 2.398

C 1.999 12.531 3.226

C 0.699 12.796 2.796

C 0.472 13.359 1.540

C -0.544 13.570 1.212

C 1.557 13.650 0.710

C 2.861 13.396 1.133

C 3.707 13.638 0.490

C 8.767 5.347 10.711

C 9.511 4.301 11.276

C 10.103 4.460 12.527

C 9.960 5.660 13.229

C 10.422 5.776 14.207

C 9.229 6.704 12.661

C 8.633 6.550 11.410

C 8.077 7.373 10.964

C 10.755 2.723 1.225

C 10.417 2.674 -0.136

C 10.235 1.447 -0.774

C 10.393 0.250 -0.073

C 10.257 -0.704 -0.580

C 10.728 0.294 1.281

C 10.906 1.516 1.928

C 11.184 1.546 2.981

C 12.429 2.542 7.009

C 12.655 1.992 8.280

C 11.808 1.758 8.924

C 13.957 1.735 8.704

C 15.042 2.014 7.869

C 14.815 2.570 6.608

C 16.057 1.799 8.196

C 13.515 2.841 6.179

C 5.011 12.009 8.264

C 6.179 12.707 7.939

C 6.742 12.430 7.050

C 6.623 13.741 8.758

C 5.911 14.085 9.909

C 4.753 13.381 10.240

C 6.264 14.892 10.549

C 4.300 12.341 9.425

C 6.570 9.800 -1.354

C 5.848 10.840 -1.957

C 5.290 10.658 -3.220

C 5.450 9.447 -3.899

C 6.167 8.413 -3.296

C 5.015 9.315 -4.888

C 6.724 8.584 -2.029

C 7.270 7.770 -1.553

C 2.682 8.991 1.194

C 3.416 8.226 0.278

C 3.110 8.281 -1.079

C 2.085 9.109 -1.541

C 1.367 9.885 -0.631

C 1.864 9.162 -2.606

C 1.658 9.827 0.733

C 1.089 10.424 1.440

C 5.772 3.282 0.633

C 4.666 2.600 0.112

C 4.639 1.206 0.128

C 3.825 3.162 -0.291

C 5.704 0.483 0.665

C 5.679 -0.605 0.680

C 6.798 1.168 1.201

C 6.835 2.559 1.185

C 11.982 10.878 1.319

C 10.949 11.077 0.391

C 10.921 12.226 -0.395

C 11.921 13.193 -0.266

C 12.927 13.019 0.689

C 11.912 14.085 -0.892

C 12.959 11.871 1.485

C 13.734 11.734 2.236

C 13.491 11.844 6.451

C 14.110 11.229 7.545

C 14.155 10.142 7.600

C 14.649 12.012 8.565

C 14.584 13.403 8.502

C 13.977 14.017 7.402

C 15.000 14.006 9.307

C 13.437 13.244 6.377

C 3.279 4.292 7.867

C 2.225 3.460 7.455

C 2.005 2.240 8.090

C 2.833 1.831 9.136

C 2.652 0.885 9.644

C 3.905 2.641 9.522

C 4.134 3.864 8.894

C 4.982 4.483 9.183

C 9.801 11.726 9.305

C 10.506 12.649 8.525

C 10.673 13.959 8.974

C 10.134 14.363 10.197

C 10.264 15.387 10.546

C 9.421 13.443 10.970

C 9.254 12.134 10.530

C 8.695 11.413 11.123

C 12.791 6.249 8.078

C 13.853 5.508 8.614

C 14.533 4.982 7.949

C 14.008 5.423 9.999

C 13.120 6.078 10.852

C 13.236 6.002 11.932

C 12.063 6.816 10.314

C 11.893 6.894 8.934

C 2.072 3.521 2.533

C 2.047 2.120 2.526

C 1.443 1.435 1.474

C 2.530 1.575 3.337

C 0.856 2.142 0.421

C 0.383 1.609 -0.404

C 0.878 3.537 0.430

C 1.478 4.231 1.481

C 5.486 8.184 11.661

C 5.003 6.898 11.925

C 5.151 6.349 13.199

C 5.780 7.068 14.216

C 5.901 6.625 15.203

C 6.252 8.357 13.954

C 6.102 8.916 12.686

C 6.467 9.919 12.474

H 20.091 5.803 2.799

H 18.498 6.572 2.865

H 19.604 8.661 2.069

H 20.023 8.097 3.719

H 21.413 7.586 0.293

H 23.789 7.688 -0.160

H 25.354 8.225 1.617

H 24.529 8.636 3.868

H 22.160 8.469 4.332

H 21.282 -1.326 1.738

H 22.233 -0.105 2.588

H 24.166 -1.650 1.484

H 22.902 -2.828 1.888

H 25.249 -3.346 3.309

H 25.915 -3.373 5.636

H 24.820 -1.912 7.239

H 23.049 -0.421 6.499

H 22.373 -0.403 4.174

H 18.577 -4.817 -0.168

H 17.033 -3.975 -0.333

H 16.714 -6.115 0.778

H 16.365 -4.783 1.869

H 18.506 -7.626 1.262

H 20.029 -8.556 2.896

H 20.549 -7.317 4.923

H 19.557 -5.128 5.280

H 18.053 -4.185 3.636

H 14.423 -2.977 0.766

H 14.116 -3.083 -0.946

H 11.893 -3.617 -0.655

H 11.823 -2.773 0.908

H 10.497 -5.319 0.852

H 10.756 -7.412 2.039

H 12.936 -8.076 2.880

H 14.844 -6.600 2.585

H 14.580 -4.500 1.413

H 16.374 5.247 2.594

H 16.507 3.819 3.613

H 14.977 4.629 5.161

H 14.354 5.830 4.017

H 15.811 7.807 3.441

H 17.198 9.526 4.434

H 18.369 9.099 6.521

H 18.144 6.936 7.605

H 16.730 5.229 6.632

H 20.476 -0.036 3.988

H 18.900 -0.843 3.938

H 19.268 0.663 6.358

H 20.506 -0.601 6.109

H 18.273 -0.496 8.319

H 16.820 -2.267 9.101

H 16.268 -4.130 7.647

H 17.178 -4.212 5.392

H 18.629 -2.440 4.609

H 16.438 -2.367 -5.589

H 16.818 -4.067 -5.308

H 14.097 -3.152 -4.649

H 14.553 -3.327 -6.364

H 15.570 -5.482 -3.539

H 15.112 -7.857 -3.457

H 13.794 -8.905 -5.211

H 12.896 -7.546 -7.015

H 13.297 -5.162 -7.058

H 15.011 7.846 -1.992

H 13.479 8.353 -1.281

H 14.577 9.711 0.325

H 16.143 9.054 -0.100

H 13.642 11.529 -0.987

H 13.994 13.288 -2.611

H 15.921 13.216 -4.093

H 17.473 11.351 -3.954

H 17.120 9.594 -2.330

H 20.665 -5.636 -2.103

H 20.561 -4.537 -0.729

H 23.109 -4.271 -1.066

H 22.968 -5.781 -1.955

H 22.476 -7.837 -0.816

H 22.539 -9.104 1.241

H 22.637 -7.955 3.380

H 22.682 -5.524 3.439

H 22.624 -4.259 1.378

H 19.188 -1.936 -5.308

H 19.090 -0.470 -6.295

H 20.061 -1.467 -8.008

H 21.385 -2.024 -6.985

H 17.779 -2.862 -7.419

H 16.947 -5.128 -7.626

H 18.496 -7.003 -7.555

H 20.871 -6.592 -7.241

H 21.699 -4.331 -7.007

H 17.012 8.241 -2.635

H 18.388 7.269 -2.108

H 19.387 7.754 -4.398

H 18.164 9.010 -4.523

H 19.220 8.468 -1.164

H 20.471 10.077 0.141

H 21.632 11.907 -0.964

H 21.533 12.104 -3.381

H 20.250 10.520 -4.685

H 14.286 4.791 -4.401

H 13.566 3.447 -5.275

H 11.590 4.500 -5.548

H 11.705 5.296 -3.969

H 13.654 7.096 -3.858

H 14.279 9.194 -4.884

H 13.605 9.685 -7.165

H 12.314 8.050 -8.420

H 11.687 5.947 -7.396

H 16.250 0.368 5.119

H 16.467 -1.396 5.206

H 14.744 0.312 6.774

H 15.268 -1.329 7.069

H 12.491 0.677 6.129

H 10.370 -0.097 5.264

H 10.104 -2.409 4.564

H 11.984 -3.940 4.735

H 14.101 -3.166 5.606

H 12.125 1.396 -4.511

H 12.082 0.831 -6.182

H 10.738 -0.959 -4.342

H 10.194 -0.289 -5.874

H 8.307 -0.575 -3.655

H 6.868 0.930 -2.426

H 7.433 3.287 -2.248

H 9.463 4.122 -3.294

H 10.918 2.610 -4.502

H 23.824 2.716 3.625

H 23.373 1.846 2.155

H 24.790 3.850 1.089

H 25.491 2.313 1.555

H 24.407 5.267 3.554

H 25.846 6.431 5.110

H 28.156 5.738 5.399

H 29.024 3.875 4.100

H 27.591 2.726 2.526

H 19.905 2.899 -8.031

H 18.802 4.181 -7.536

H 21.397 4.560 -9.005

H 19.791 4.726 -9.683

H 22.005 6.145 -7.052

H 22.035 8.498 -6.483

H 20.373 10.002 -7.419

H 18.675 9.133 -8.930

H 18.650 6.784 -9.498

H 15.532 3.681 -6.302

H 17.052 4.116 -7.127

H 14.551 3.036 -8.244

H 15.671 4.283 -8.851

H 18.144 3.135 -9.259

H 19.140 1.505 -10.744

H 17.780 -0.257 -11.716

H 15.407 -0.379 -11.188

H 14.409 1.260 -9.713

H 21.032 6.331 -3.120

H 21.714 6.474 -1.522

H 22.839 7.506 -3.791

H 23.715 7.133 -2.298

H 25.551 5.680 -2.409

H 26.924 4.094 -3.616

H 26.196 3.243 -5.773

H 24.057 3.950 -6.688

H 22.682 5.529 -5.475

H 8.886 0.605 5.703

H 8.772 -1.041 7.473

H 4.813 0.100 8.278

H 5.117 2.478 5.717

H 4.635 2.371 7.393

H 4.046 1.199 6.238

H 7.654 -1.873 8.864

H 5.939 -2.045 8.577

H 6.518 -0.869 9.732

H 9.078 5.408 -3.152

H 10.737 9.906 -3.900

H 11.248 9.893 -1.987

H 11.779 8.390 -1.270

H 10.211 9.046 -0.865

H 8.912 6.412 -5.352

H 9.270 8.008 -6.457

H 10.709 8.946 -6.138

H 9.139 9.584 -5.713

H 6.035 15.644 4.397

H 7.162 14.854 5.473

H 6.202 13.905 4.364

H 6.743 16.683 2.879

H 9.342 16.830 0.522

H 8.545 17.841 1.703

H 7.597 16.878 0.595

H 10.261 15.208 1.125

H 10.273 13.349 2.679

H 2.160 12.111 4.155

H -0.100 12.574 3.411

H -0.521 13.996 0.231

H -1.002 14.260 1.890

H -1.109 12.661 1.193

H 1.392 14.056 -0.224

H 3.349 14.055 -0.428

H 4.265 12.748 0.285

H 4.338 14.348 0.982

H 9.620 3.412 10.764

H 10.650 3.687 12.938

H 10.934 4.874 14.469

H 9.666 5.982 14.936

H 11.120 6.587 14.178

H 9.129 7.596 13.170

H 7.695 7.071 10.011

H 8.726 8.215 10.838

H 7.264 7.643 11.605

H 10.302 3.551 -0.668

H 9.981 1.424 -1.774

H 10.006 -0.530 -1.605

H 9.468 -1.250 -0.107

H 11.164 -1.269 -0.526

H 10.845 -0.586 1.808

H 11.274 2.564 3.297

H 12.120 1.047 3.117

H 10.431 1.057 3.563

H 10.898 2.025 8.428

H 11.891 2.309 9.837

H 11.803 0.710 9.140

H 14.121 1.336 9.641

H 15.614 2.782 5.990

H 16.746 2.079 7.427

H 16.153 0.752 8.397

H 16.270 2.354 9.085

H 13.355 3.262 5.250

H 6.248 11.621 6.553

H 7.728 12.126 7.333

H 6.803 13.270 6.390

H 7.483 14.256 8.513

H 4.228 13.630 11.093

H 7.158 15.310 10.136

H 6.468 14.508 11.527

H 5.512 15.651 10.613

H 3.446 11.820 9.677

H 5.730 11.740 -1.465

H 4.754 11.424 -3.658

H 6.286 7.515 -3.791

H 5.239 8.333 -5.248

H 5.424 10.042 -5.558

H 3.954 9.440 -4.831

H 7.289 6.923 -2.207

H 6.785 7.503 -0.638

H 8.272 8.085 -1.347

H 4.184 7.621 0.609

H 3.645 7.706 -1.748

H 0.615 10.506 -0.969

H 2.514 8.500 -3.139

H 2.014 10.163 -2.952

H 0.847 8.875 -2.774

H 0.354 10.997 0.913

H 1.750 11.085 1.962

H 0.603 9.779 2.141

H 3.825 0.705 -0.261

H 3.077 2.482 -0.642

H 4.161 3.773 -1.103

H 3.411 3.783 0.476

H 4.771 -0.947 0.229

H 5.727 -0.949 1.692

H 6.515 -0.989 0.133

H 7.583 0.639 1.611

H 7.647 3.058 1.581

H 10.206 10.368 0.290

H 10.158 12.364 -1.076

H 13.652 13.744 0.807

H 11.075 14.045 -1.558

H 12.818 14.132 -1.460

H 11.834 14.954 -0.273

H 14.391 12.579 2.229

H 14.291 10.847 2.016

H 13.283 11.641 3.202

H 13.693 9.721 6.732

H 13.639 9.808 8.476

H 15.177 9.829 7.647

H 15.100 11.557 9.374

H 13.929 15.047 7.350

H 14.858 15.043 9.085

H 16.046 13.801 9.402

H 14.505 13.766 10.225

H 12.997 13.701 5.563

H 1.611 3.754 6.679

H 1.226 1.636 7.785

H 1.796 0.408 9.215

H 2.476 1.066 10.684

H 3.507 0.251 9.532

H 4.533 2.330 10.280

H 4.990 5.375 8.593

H 5.890 3.941 9.022

H 4.899 4.741 10.218

H 10.903 12.360 7.617

H 11.199 14.635 8.398

H 10.832 15.939 9.827

H 10.782 15.382 11.482

H 9.305 15.845 10.672

H 9.016 13.736 11.873

H 8.680 10.470 10.617

H 7.692 11.762 11.256

H 9.163 11.298 12.078

H 14.254 5.169 6.933

H 14.483 3.931 8.146

H 15.532 5.328 8.114

H 14.785 4.871 10.394

H 14.093 5.402 12.157

H 12.363 5.549 12.353

H 13.366 6.980 12.346

H 11.406 7.305 10.941

H 11.102 7.429 8.542

H 1.429 0.403 1.472

H 2.417 0.523 3.177

H 2.076 1.845 4.268

H 3.571 1.822 3.363

H -0.007 2.315 -1.108

H -0.415 1.004 -0.027

H 1.106 0.985 -0.887

H 0.447 4.060 -0.348

H 1.484 5.263 1.484

H 4.538 6.357 11.179

H 4.791 5.401 13.391

H 5.479 5.642 15.207

H 5.401 7.231 15.930

H 6.942 6.567 15.443

H 6.714 8.897 14.702

H 6.259 10.166 11.454

H 7.523 9.957 12.642

H 5.978 10.620 13.117
