# Supplementary material for: Structure-conserving spontaneous transformations between nanoparticles
Source: Nat Commun. 2016 Nov 10;7:13447. doi: 10.1038/ncomms13447 (PMC5110647; doi:10.1038/ncomms13447)
Supplement: Supplementary Data 2 — Coordinates of the geometry obtained from DFT optimization of force-field global minimum geometry of the adduct [Ag25Au25(DMBT)18(PET)18]2-. [file ncomms13447-s3.docx]

Coordinates of the geometry obtained from DFT optimization of force-field global minimum geometry of the adduct [Ag_25_Au_25_(DMBT)_18_(PET)_18_]^2-^.

698

Au 25.194461449881004 19.544065655610552 18.487687728342909
Au 22.757506788350312 22.030626197534929 18.534221329025343
Au 19.988281891814644 20.155941978205050 16.455830859934068
Au 21.709911811833184 17.265332737917401 15.141359717078668
Au 26.115878259125296 19.250360123998654 15.513536311349199
Au 28.034684951730220 20.637512302484240 18.421293401032830
Au 26.446687746551216 18.798139822540268 21.459057379008549
Au 24.234900023018607 17.155919799198653 19.756656674553131
Au 23.083090387105997 19.771308936330300 15.716765429123594
Au 25.148908909489936 20.738878139278182 13.098463784138143
Au 27.023736893382424 17.338017836862644 12.489597959407604
Au 26.408660574821578 16.029068670361465 15.395754783121447
Au 23.491486682248052 15.080899683376218 15.558650805495740
Au 25.037172126786466 12.372439449849058 15.129168634356175
Au 28.231855757796538 14.163049912294618 16.689651091910644
Au 27.110619469248601 17.663731522043623 17.760719227316681
Au 22.493097703128239 15.572005993867300 18.210642936729332
Au 19.805609671562895 14.858136058548464 15.188082769789958
Au 21.321046920738731 16.035318893495145 12.259742836630497
Au 24.401861140994395 17.570132679617188 14.035196058735767
Au 25.409945542883257 15.046444050689439 17.951941393405217
Au 23.622777375367210 13.862880471964871 20.554145977055470
Au 21.124460010111939 16.659461657239738 20.742987463571662
Au 22.012874214489919 18.511168185943173 17.931117308902945
Au 24.332803419979250 17.411907064944682 16.868383569487214
S 25.128464104116254 22.082881774067886 18.769367418200172
S 20.376359661250735 21.994644678376197 18.038366709427336
S 19.444442261964401 18.483946292557629 14.801336443011024
S 28.343876347076648 20.204202153173092 20.872541110307971
S 24.846275501011615 17.171114385283364 22.305070127519837
S 28.137298481334852 20.724053189121673 15.979133496111052
S 23.188680124487714 21.805947719841498 14.171575158630651
S 26.853474460671048 19.608966182668606 11.713856701597212
S 27.407631353007201 15.039518307820686 13.184621953784184
S 22.624090872899359 12.726986516378005 14.931049414003780
S 27.459652756930033 12.164166512443863 15.391424739877500
S 29.160477391652009 16.106675515594400 17.916592509678857
S 19.278321005643743 14.777926619640841 12.729066012311943
S 23.390449245594425 17.231337608201425 11.731395298293945
S 20.019789639298349 15.224199184488491 17.579653778003205
S 25.578377970716168 12.887420136540223 19.353041258751414
S 21.539544529158704 14.458766159833454 21.634361931756889
S 20.627058252289057 18.906241677179231 20.009034667663382
C 25.411705850903903 22.486832561827939 20.630248381393365
C 26.186210025106966 23.855680903228656 20.713542973548172
C 27.652935321103900 23.703715232800814 20.233970706618653
C 28.040308315064678 23.373210470389928 18.875777668399301
C 29.428191393239885 23.101037840455017 18.529324086529336
C 30.447005200369095 23.112549547951406 19.568096501446107
C 30.074595587415867 23.486139199149751 20.928594136305271
C 28.689836332211108 23.781165863978806 21.250108588215259
C 28.483750833761693 15.533286539441056 19.635410197525690
C 29.160726499261120 14.191139132221029 20.043214794102717
C 29.352134680132856 14.050558868514747 21.575302119938346
C 29.836725039940308 12.763168141970016 22.054952851181884
C 30.167390539545160 12.578461804432907 23.459268334109250
C 30.044041682234187 13.701428129645691 24.391619282111886
C 29.573120513892693 14.990004878185387 23.899403072379162
C 29.221785508314454 15.165364132027355 22.499510402853222
C 24.402366381818446 11.819228828934099 18.217306642236853
C 23.479635515934167 10.953145100635419 19.141111455914693
C 24.363316374123578 10.164343979715300 20.118736344778096
C 25.321951580802850 9.186865629220982 19.633936897273610
C 26.248865470461801 8.575988321810746 20.569026395203707
C 26.203374665856536 8.959934268402401 21.976226095626672
C 25.289766067733208 9.991775968495340 22.443228348159334
C 24.364425549585601 10.590743822485376 21.496919523097823
C 20.105211970970892 13.447661957054217 18.280505975649344
C 18.747429994235951 12.693925821887149 18.508346384508336
C 19.051236861526803 11.407117227537531 19.330745931286369
C 17.998629558983740 10.592482683963720 19.909764925124168
C 18.345609945218175 9.439510602453066 20.728946843397487
C 19.747347093017218 9.103617276945458 20.979899917078651
C 20.809646606919529 9.898018699388537 20.370298864762056
C 20.433528259642891 11.027154724479050 19.542278292746378
C 22.024606153857420 19.898892365073639 20.940217168835837
C 21.266484719299140 21.015539328081193 21.740970467815668
C 22.218060157479940 22.151155568918174 22.156038796096453
C 22.180621531696314 23.437717112619790 21.466845903253638
C 23.111972013307994 24.490920924552814 21.835799598212699
C 24.061939262692107 24.262319119698418 22.901979111376043
C 24.122287213005752 22.969140511370799 23.571823187905260
C 23.220535099940506 21.902296702999259 23.181438277692795
C 25.956695827394743 15.569639297825367 22.184090041552263
C 26.396298271222289 14.952610293482291 23.568171889886248
C 25.255683898532375 14.132346709473351 24.219096639150546
C 24.677171547660915 14.535879106444410 25.483965193164892
C 23.560445228224825 13.796764790011599 26.051876445676818
C 23.052078340286535 12.621902511675241 25.363756407640484
C 23.680349673585262 12.175668466383454 24.129488020032898
C 24.769332927207074 12.937189795906352 23.548313808813276
C 22.683672240058019 12.960800779759285 13.005681064110435
C 21.418096615379646 12.341198906262392 12.350321839861385
C 21.395605263050641 10.802048799499868 12.436885446672697
C 22.396695371392862 10.038552191356684 13.158408291909174
C 22.346895677023895 8.582240023950675 13.130843463192104
C 21.330578729798312 7.885196925457218 12.348822230655983
C 20.333558020892600 8.661216639081417 11.607337203596526
C 20.370312746052036 10.115176682424595 11.675573970395767
C 20.623749036104368 23.426067641380961 16.754831702452982
C 21.032004564157457 24.737973725851823 17.518624066464991
C 21.397559759363958 25.838246404544122 16.505095109274151
C 20.383239718390527 26.741553723709345 15.995639550794605
C 20.690522013032972 27.604681798790537 14.870674346784199
C 22.019573976666052 27.574635524920478 14.267474078207785
C 23.063399228906050 26.737406365727907 14.849760240613678
C 22.741298687769767 25.871837090605940 15.969506914534763
C 27.453398822110678 11.040234981413574 16.966606671316217
C 28.945490028294934 10.779249885927511 17.375423855531132
C 28.973467395824862 10.016311186374370 18.716975931850644
C 29.510182619048550 8.668085612134263 18.774352536194833
C 29.612708625254879 7.972704997029115 20.052975993530922
C 29.196357302902182 8.653011363396915 21.272469945056002
C 28.597141191348740 9.975246143053004 21.194820356452251
C 28.444167476902841 10.637071327708103 19.917612691709991
C 25.674415793810262 14.386739813243066 12.591496130917781
C 25.615976790225542 14.175996444059040 11.022790258135725
C 25.190391277007762 12.710370945244875 10.765473349937388
C 23.839756063663987 12.377299825717547 10.355474566282066
C 23.370192619759713 11.003917044472708 10.428151646141069
C 24.260790936596802 9.952340829186097 10.904321231531119
C 25.642745817647054 10.274937890998247 11.252980451514189
C 26.094764203470071 11.657700064903988 11.197677923503285
C 24.108974225543264 22.944249397422354 15.440086188739086
C 24.783639529587518 24.088134232992545 14.617287305094653
C 25.680895873979029 25.043135288747944 15.436333146488344
C 25.856309560200732 24.861587202369954 16.862412650702716
C 26.839973696644019 25.645643629305823 17.593763795247881
C 27.672882185550861 26.615028272249155 16.893062852650747
C 27.450838845078131 26.846456635822914 15.470787548789463
C 26.445975381845027 26.074904320955632 14.760528225040224
C 19.430340277588471 19.572462514902718 13.195695042446737
C 18.284353452331111 20.665245742652246 13.264476567788982
C 18.824102291256885 22.109529008559456 13.075223437445818
C 19.860402795961924 22.623378329679912 13.960802766869614
C 20.332423295122275 23.997521019009589 13.843983042028706
C 19.707181902846621 24.895976550684132 12.881296850480812
C 18.691090042823369 24.382181088090299 11.967131831302783
C 18.283819533430375 22.982441377686932 12.048250368214390
C 22.049338788765500 15.029153413326783 23.420644961169693
C 20.801651177426130 15.073787574404282 24.417568479654953
C 19.638817263182567 14.205097912916015 23.860306786936217
C 18.335208086735779 14.783305209344189 23.581149220054964
C 17.389111235822181 14.050123770593393 22.744991605154993
C 17.773369056532811 12.780515629554438 22.135099649527341
C 19.061844297530737 12.167982093242159 22.460716362671139
C 19.971615721159711 12.877938660616804 23.349779386559671
C 17.972698311211428 16.198006137230625 12.827388646424911
C 16.611006177855348 15.653028372216880 13.392867155917674
C 15.746219036733361 16.795604946767092 13.999222720639240
C 14.585579352322362 16.437800417903709 14.808127542789531
C 13.910639667101169 17.424289549107588 15.639613904977892
C 14.455755315118592 18.775089490995292 15.696472255795916
C 15.602994508006491 19.133380176164493 14.896123012746772
C 16.213989811435528 18.170824725503063 14.000304972757890
C 29.520549780345956 18.714812554660849 20.409801749368686
C 30.792555312102497 19.213362357920403 19.635293353280076
C 31.833658298703273 19.887545020263321 20.559195673826935
C 31.446282745604961 20.957776673903261 21.465302284770864
C 32.443522939636182 21.594210232319675 22.309704965889441
C 33.830924710562812 21.140682908589394 22.287037754012776
C 34.215490491218915 20.042247850646657 21.403275137887388
C 33.213969002323850 19.423926598387254 20.539787407295709
C 25.818723427665162 19.409207090908101 10.089610338979940
C 26.244381844916251 20.571738547881520 9.099979912643295
C 26.203304840858831 21.901769340492685 9.890404292948558
C 27.386464571569860 22.346592490321022 10.605192355934104
C 27.306307698749620 23.467723469490366 11.529867104447710
C 26.024025330663644 24.112211783241673 11.754850144814494
C 24.817390853570835 23.613612230111613 11.110627396755486
C 24.917313838660892 22.509433184665578 10.173650042559199
C 22.589819088970525 18.997798257331727 11.783293715449279
C 21.589581478265114 19.187324159122984 10.576758473150306
C 22.241617031241663 18.569497010078031 9.311301195748875
C 23.243778497569526 19.298310285980474 8.558007189414456
C 24.086459615580086 18.611905880770035 7.584707221364431
C 23.950293291536912 17.170830141883510 7.387663942305606
C 22.933058394090523 16.430445300025184 8.137827420899955
C 22.072100542420273 17.139779975374445 9.078458083429846
C 27.336162570167282 22.427665355831213 15.503519462145082
C 28.429145184089613 23.457949527734527 15.017318693014518
C 29.455116040157648 22.662838718254974 14.168030797253611
C 30.872598066509454 22.703526137103474 14.469771897450855
C 31.795560466308658 21.808389295568016 13.772994639241313
C 31.285616584332466 20.844478538721130 12.800545474416403
C 29.851527138231152 20.792607309498823 12.516584175632818
C 28.952272953952420 21.692795090475723 13.209440411853146
Ag 14.455238204164223 26.502542690024743 22.193583888577223
Ag 18.308955483941190 27.470532049272514 21.592069773015982
Ag 20.599386701452570 23.629134375742417 23.354285399120311
Ag 16.493978680281078 22.355818415992896 23.470304531631349
Ag 14.246743520620761 24.650834833870320 19.824342491776942
Ag 15.964696702055351 25.091149624438774 24.386477827506990
Ag 12.719756735760338 25.842790728509627 27.571482009950540
Ag 9.849317237912503 23.471908797029386 26.624681843634416
Ag 11.690504651890523 22.502566224989710 23.576988401808826
Ag 14.061633961492429 20.822234010844006 22.788734685930784
Ag 12.122901591401554 18.983698100507567 20.873922868311141
Ag 9.625580572328753 22.901220402419629 21.049415359365646
Ag 11.911108371467330 25.072723934620459 21.727225237682944
Ag 15.510664320595280 21.935504773998552 20.477196245047708
Ag 12.605773379381285 22.229070099417598 20.457297176103591
Ag 14.886351485210666 21.396869409453206 17.484717433229068
Ag 17.707320594908762 22.993052221666211 18.571051018747763
Ag 16.636671220163681 24.571550362996586 21.624373577670436
Ag 14.142356515978094 23.613394933959999 22.565174410694855
Ag 12.819666460658476 28.487660149329017 19.408012330591539
Ag 10.852427040791893 28.127439642636521 22.971684414991234
Ag 12.636712802231990 25.106559665908705 24.531139410372766
Ag 15.950588667813374 19.136538434334081 26.036815920682496
Ag 14.163011367649183 22.625935977919823 25.322072998987402
Ag 17.222011859672140 19.616625537584813 22.437104173700650
S 16.191074551435133 28.485010869852665 22.560408746489468
S 19.480179395500819 25.929245090780594 23.295662425549548
S 18.898171871612380 21.726284569234789 23.874413395412823
S 15.132025894449923 25.618707310626391 26.781099208101470
S 10.377258676520457 25.141030472877091 28.417205533043578
S 9.294992216941068 22.107424418087618 24.593085981003984
S 14.447188024005504 18.402050538753262 21.880729187191353
S 10.032046581333725 20.471057813252919 21.508396629657820
S 9.406631964232849 25.401724786656843 21.200002623660922
S 12.385669334371004 21.125010299595527 18.011844528239809
S 14.304732813773263 26.852061344172839 18.378678595778432
S 10.639681361655503 26.819751172676821 25.125232980330157
S 17.142962643258421 21.819398160348342 16.367723625695575
S 18.878916330304804 24.354178277433707 20.352392144066997
S 14.392623416562325 20.708373317395161 27.049293948173325
S 11.175011318722634 29.535616005954690 20.920405164918577
S 17.496726958375504 20.209666982075685 19.984395391772985
S 17.477211245248160 18.109362132082726 24.492056740622051
C 14.164242266263953 17.467923643010415 23.456876626589757
C 15.103172963196215 16.391993213999886 23.758101022184295
C 13.050974294339055 17.726231072666316 24.364128597500091
C 14.949025780886648 15.567197440359891 24.938809484445947
C 12.914188691853919 16.910712484700486 25.555319464098908
C 11.971472192380622 18.791104537584744 24.118721732606133
C 13.837883463141813 15.830717146391960 25.845539167502391
C 13.573055704144089 14.925036596071921 27.080401714257274
C 16.590298888306492 22.851849970829981 14.901317950762721
C 16.973828961811435 24.246237630022122 14.760564188448084
C 15.753559452818564 22.259893720157724 13.864554041642601
C 16.542719760991297 24.996777188075590 13.601224423756962
C 17.866175580799151 25.037210174004329 15.730301496385781
C 15.366882252019535 23.017208922630722 12.696002613171050
C 15.770202759316597 24.393771881955715 12.548521477149096
C 15.524667945018106 25.146403690701810 11.223405135004766
C 15.629848468970453 29.493581112154079 21.114952254663798
C 14.509700413215086 30.420642971896939 21.253678911110001
C 13.808277103629838 30.650752561705414 22.616204134328630
C 14.116287497776579 31.218003121025490 20.115191690630201
C 14.787080233160774 31.101493077227726 18.839826114030597
C 14.406648471713657 32.071786662182951 17.695845031834700
C 15.894559390385439 30.174341581629196 18.694838272415090
C 16.323177262566869 29.398402887733923 19.838891869971746
C 9.460477301603181 29.155210683212552 20.301662097581126
C 8.378337983819637 29.340066040987928 21.270794200213782
C 6.997737818477769 29.435750722347027 20.842683712367847
C 6.677108054218115 29.267585589679886 19.436628638005189
C 5.200186808114781 29.213925127871896 18.984506305473808
C 7.731384046620353 28.935878438934083 18.506031683409873
C 9.123474019071285 28.878381949400136 18.902321232933851
C 10.164578038676149 28.468937732547687 17.834706022626339
C 15.194160752615000 20.899959989797967 28.696030072714578
C 16.105312645612699 19.873385115152367 29.189425433202715
C 16.724512479422714 20.008732345895769 30.490925585820452
C 16.491711948276492 21.192116327810069 31.292886279711563
C 17.164915781023478 21.334994207306501 32.672178738124309
C 15.668630632090620 22.246501166677742 30.756232613969715
C 14.986578357671229 22.098867865910716 29.494485716776499
C 14.281214841144433 23.320325854820396 28.910396088869110
C 17.300558005352649 18.516017389446549 19.160836827130300
C 17.022576142694327 18.493583652980266 17.732515328845970
C 16.871560001287634 17.253059206595161 17.009022141585323
C 16.949183652583937 15.977891831625316 17.679855836790715
C 16.713993674097448 14.679006184195844 16.875835026942088
C 17.280986322007497 15.986846427301886 19.082467825042382
C 17.487893672694852 17.216745192870881 19.824978674224507
C 17.839848702967252 16.984083920505800 21.302192655846795
C 19.252581170584779 18.493400792014551 24.826639114938985
C 19.774623418542479 18.515108936632455 26.191860875141590
C 18.876527947543160 18.824369995321565 27.418203230730320
C 21.169430428514037 18.196017379838977 26.378729228162420
C 22.051658887154773 17.939965134924527 25.262133046230886
C 21.579949553411307 18.192425011298280 23.921150681275385
C 23.331591888392680 17.125836093379526 25.509994027898141
C 20.184148893212285 18.473656949287413 23.708070073122723
C 11.574253032136857 27.924932050943379 26.318904927796861
C 12.885251685654287 28.486402874437879 26.014906401017381
C 13.638970951051027 28.184727682395266 24.722479751192939
C 13.570028984883939 29.314687185054147 26.988627487214345
C 12.985054930459334 29.590398514088811 28.280532163414378
C 11.691636548320005 29.012902583588339 28.608920386619097
C 13.761586320174704 30.426362331898318 29.329675810459488
C 10.980466112402238 28.216413862932047 27.624882208541532
C 13.531595028318142 26.519257880370326 16.735330803966125
C 12.575205876463372 27.413535261492893 16.094188296040588
C 12.067966053966686 27.124277818561286 14.766060270393295
C 12.504500917366844 25.934736372431598 14.050991740442996
C 13.414817162220848 25.017422284400045 14.706094128233698
C 11.952977369468162 25.581494520768761 12.645421506304123
C 13.914644431163794 25.305936998470678 16.031689807830780
C 14.717762154010273 24.210637134307227 16.724686626628685
C 9.606192930134732 25.545124456741675 19.337007934587348
C 10.810422899669573 25.199241589116504 18.596170217253142
C 10.791513797091740 25.158262543581365 17.143334666367274
C 9.587641453417717 25.547984120406888 16.431090822368155
C 8.427861422399618 25.993213689627808 17.174589467369447
C 9.512845695194713 25.480503805340280 14.884142361197540
C 8.409427926782099 25.950233908175392 18.617004142502260
C 7.123414120955269 26.284754970872317 19.406279662260630
C 12.647003829634150 19.309516979689768 18.433406679648602
C 11.605734390643230 18.318800019405558 18.146079183590210
C 11.909116292670507 16.905785278108056 18.262928806528159
C 10.248038986996962 18.749465851839798 17.549218462032115
C 13.243952475335030 16.446738934721679 18.568561535883816
C 13.654067547646804 14.963161435550647 18.415871173077370
C 14.276445488732156 17.418712023828306 18.853424511076437
C 13.964540220657501 18.822450182125845 18.825946204125039
C 18.710834149899853 26.022426310123883 19.521385162003767
C 17.544422434599664 26.336996148172119 18.710447932189098
C 17.500087787932284 27.504145054072310 17.867795931895618
C 18.632086039267950 28.398669768125611 17.823793526151814
C 19.797157020211070 28.106275495037501 18.634007278628140
C 18.694211077906907 29.542496551331929 16.788483701022329
C 19.860332529741093 26.930780476768685 19.481527544520532
C 21.175931440646959 26.526009008416608 20.178989153813170
C 19.995253666538794 27.093329643264035 24.639076889030584
C 21.035979429569650 26.744631087541904 25.619369164425674
C 21.914760364781991 25.467134663465998 25.467204639513987
C 21.260819564485104 27.610394329868150 26.772272592374350
C 20.517142179469428 28.848830201649992 26.931111480170891
C 19.580182739591606 29.240421226337727 25.896384862061339
C 20.563901983818592 29.744192934798040 28.198459578223527
C 19.291496151322601 28.368582755911479 24.777722460233743
C 9.361135847880664 20.326938802645021 25.179654301191121
C 8.688785673346336 19.301671798180276 24.369469876967393
C 8.727471201589120 17.895765193424904 24.747829791601983
C 9.381456722720705 17.481386649195869 25.980473860413969
C 9.534040651320739 15.987663562115928 26.374401382260274
C 10.041236963098861 18.492128620328710 26.770471474008822
C 10.098307898120870 19.882372883683882 26.361534213761022
C 11.311440848677151 20.621597088523643 26.947996842511021
C 15.777565162173897 27.318642504442316 27.035974739148820
C 16.396504563923614 28.036445776176375 25.939289076293012
C 16.829291697997185 29.402383258145637 26.111918369758818
C 16.856693835037888 29.986648432088959 27.437399837894176
C 17.441852737068839 31.392643378235356 27.720155336311663
C 16.318664813786608 29.233873323190487 28.548684043908533
C 15.683653989168622 27.951718147334816 28.351695866644281
C 14.847988379251047 27.326931639695349 29.490781702801808
C 18.928182537679174 21.693061465118124 25.729576485079431
C 20.189502440392328 21.393077785836539 26.394918192448571
C 21.459672011791930 21.079466487379314 25.578962977278351
C 20.259581657303482 21.408184821412451 27.838671302818319
C 19.112333089036714 21.782221190144803 28.637306654067576
C 19.193078092399443 21.848816239034708 30.181190946689316
C 17.856827585383439 22.080387811728759 27.975293526316083
C 17.749485590623916 21.985359593318005 26.530622996441053
C 8.822737973383553 19.702316954427754 20.348704087593418
C 8.707692964272328 18.245122554905535 20.352030369641742
C 7.927740773044166 17.598331509595205 19.320434017203340
C 9.508790145737029 17.386169654960746 21.363515863173564
C 7.250924027405517 18.362304003615538 18.291683159927913
C 6.673168805550640 17.626561065248659 17.056883890889100
C 7.274459170168909 19.814170111006728 18.358141547983220
C 8.041977097094128 20.485374057782270 19.395167453758908
C 11.256457594439871 24.097228944622039 29.700804349392399
C 11.360027702269182 22.654258552060607 29.530858559947013
C 12.165343128575616 21.855572442560170 30.424590715582717
C 12.795297440844022 22.466089399342884 31.582145693695377
C 13.579337645293968 21.578541977103438 32.583071333327418
C 12.687235126630689 23.897710382016026 31.753090887114034
C 11.968393566440142 24.730642466050185 30.805681685245705
C 12.072292555373325 26.271548485980883 30.938513090378322
H 26.031657294635245 21.619086873283990 21.135772066391205
H 24.378229162827505 22.571392026776476 21.159896182767703
H 25.577493162049077 24.687369446121945 20.108831715426550
H 26.212148602674706 24.202420869155656 21.846910756069978
H 27.228383403666058 23.337300899724507 18.060252504212453
H 29.722306807128632 22.820665368442199 17.423690881624921
H 31.569260928812568 22.760904646640299 19.356514837572142
H 30.891796305348276 23.480231014657235 21.784162909806298
H 28.376617163357231 24.069012802685144 22.364838539189165
H 27.321833568294608 15.446426719324954 19.590291781332230
H 28.788639794774209 16.375034615830266 20.384273574278783
H 30.261359140935927 14.138912923981113 19.562105603946584
H 28.575976291042810 13.271831709736009 19.593017721442468
H 29.991903481504011 11.886389586973284 21.272172187810295
H 30.599723220773623 11.526362399184642 23.832773472388318
H 30.358252653120328 13.575520357084155 25.540973392364311
H 29.463696412647245 15.917251782886241 24.645015747955412
H 28.793312320741382 16.210062184100781 22.173407958913991
H 25.044290725616960 11.121127202618126 17.522557482898851
H 23.783793643875384 12.555608706248146 17.548448476895345
H 22.787697680875880 10.239442765749150 18.484428998798418
H 22.816965908325670 11.658235595034069 19.783507664112779
H 25.372923523397255 8.933580143900027 18.470036354742735
H 27.117638598959552 7.878192492291937 20.162795195087426
H 27.003337097639093 8.514128820265185 22.727430773695140
H 25.356077193409266 10.406474194295342 23.553594606662948
H 23.690794103871887 11.499810739293874 21.748804693992703
H 20.624242387846753 13.533557452627157 19.329470088399532
H 20.791475592085373 12.788019781396745 17.580181208124195
H 18.211024284340873 12.412283536450518 17.479308040745178
H 17.982259606367627 13.358519497626075 19.124282008689928
H 16.860258356413841 10.885228422370616 19.723950087239587
H 17.481184153501800 8.776803455305144 21.228390696851569
H 20.023446646239776 8.185558790759485 21.697658941686885
H 21.956403215544420 9.629908324448948 20.558513333165113
H 21.251989039674751 11.638969228011762 19.007071555750699
H 22.730545828629324 20.364664645183190 20.137603338455033
H 22.678778699899180 19.228414573230999 21.655657646960865
H 20.763476049968929 20.530171367500589 22.685063977102015
H 20.394526965178358 21.456971026849274 21.073737396726862
H 21.412228771813673 23.580622294431308 20.606067552019105
H 23.064346455755182 25.547293173919368 21.304330683532687
H 24.801650487016765 25.133429943526128 23.224189735580687
H 24.914473610129846 22.784666543497792 24.448282860048661
H 23.217803601891564 20.853222994197864 23.741601866871239
H 26.892864987573141 15.794638706821267 21.515392214310744
H 25.304735705854046 14.776885234353951 21.627304420464384
H 26.768083175029417 15.788737165937622 24.333991790652021
H 27.317932019643006 14.221464399600654 23.372494478537483
H 25.073394923635391 15.523832585870144 26.001138577698462
H 23.025347955020578 14.184628681276118 27.043972253733241
H 22.101407694033323 12.037593669742884 25.784640748461200
H 23.267472906303318 11.193938335064027 23.619733311038356
H 25.273210804614500 12.628719277116634 22.539780821321830
H 22.745266012496963 14.098563993554452 12.755571086927960
H 23.629212946452132 12.425327326305068 12.588419984775516
H 20.432839698326923 12.742663695272871 12.866735189059559
H 21.339433875747826 12.646902121331388 11.204291214982627
H 23.251067081457386 10.577025162233088 13.753754712363872
H 23.166279945079335 7.951013837242692 13.735076556395370
H 21.296100423054519 6.687654214727814 12.323686386726775
H 19.478465742555564 8.110536381059758 10.971527478824635
H 19.586282540346861 10.781104157879389 11.069057416688006
H 21.455935345539217 23.099738339951674 15.997490863579758
H 19.637613290217629 23.563912108190966 16.153543412120694
H 20.147803751097083 25.113386728894501 18.176621936685660
H 21.967230559946405 24.489015929092005 18.178550761748568
H 19.335137924628398 26.745363122394544 16.499072888541590
H 19.863658786994900 28.333741979567925 14.429790825520167
H 22.280431163298612 28.275131847257821 13.333205803721414
H 24.172168610155076 26.757491647223272 14.435758225965900
H 23.573390211062296 25.215233841382585 16.463305180635327
H 26.901138014313446 9.998950419273713 16.772449971738133
H 26.925306424472730 11.587466422006035 17.869711741752756
H 29.489802436219790 11.828769631353602 17.480787502736270
H 29.525933031091661 10.136099544982683 16.548700280620324
H 29.864980646441087 8.160709919903868 17.752806985205392
H 30.039007567601640 6.854746459580853 20.088759651911712
H 29.278371139229652 8.082390950915894 22.316487144235072
H 28.160275063106390 10.520580770606552 22.150365916493421
H 27.821367202457935 11.637897699827803 19.863593484103138
H 25.505696194504065 13.363011265925804 13.130458668675452
H 24.859193092564833 15.117901537666853 12.983963815361351
H 24.852752917994142 14.928398886306313 10.495430492559112
H 26.700205319433589 14.357905039445148 10.545761694288418
H 23.108719921059347 13.275793377419870 10.103327501294034
H 22.243544446788352 10.712810469514194 10.215241695390540
H 23.823312270679605 8.857966580660388 11.069451284995781
H 26.396002875625999 9.412885736209748 11.609280627781279
H 27.188195572152448 11.966969279634620 11.564696796389594
H 23.338063361675232 23.325456452709165 16.236264136126184
H 24.892216425989417 22.292054081906592 16.010449322108386
H 25.437426277881556 23.581375655099158 13.791128899333915
H 23.944678998421217 24.684208792109217 14.021595456180345
H 25.261615993109942 24.022399107289587 17.438539875594032
H 27.055029264517135 25.459796736440442 18.741104944756714
H 28.512196947793477 27.227014147066452 17.493338832346296
H 28.105141460230321 27.666262524966058 14.894077925566416
H 26.310833917776957 26.311743605333064 13.612870496054949
H 20.452356362566782 20.131612968022953 13.012253433208985
H 19.244557453508769 18.851770790251944 12.276598689140512
H 17.419874546171421 20.502323299229730 12.468887022833998
H 17.776456854136363 20.622427742701134 14.316445148357452
H 20.262622274649146 21.904061372715184 14.783252844669653
H 21.139327960487027 24.479302129680516 14.562421132672505
H 20.027937778966557 26.044999137480627 12.901511048331635
H 18.139402567408354 25.113345630360261 11.200984483163971
H 17.422092084801296 22.570482806382081 11.348658470866757
H 22.551783325605435 16.085837455882405 23.345357720871839
H 22.842548390302188 14.264986314911203 23.789988770357137
H 20.421700242200089 16.183982772835947 24.588045231222416
H 21.182393356567385 14.641685459505766 25.468342674559342
H 18.089088716293759 15.876202866961071 23.983246452830372
H 16.328386910097144 14.507449650714205 22.479744697364492
H 17.038943574529714 12.237430407603922 21.375138954880928
H 19.396424348469541 11.153449788642378 21.962833578686066
H 21.049110847287789 12.436385151725094 23.575022778009895
H 18.420902349498615 16.987294005159651 13.569337459421181
H 17.802738008566248 16.695246039767500 11.755543599401031
H 16.823584908575491 14.875635657746484 14.266061641835211
H 15.982198499041047 15.069139313970776 12.551161086942576
H 14.203135655405799 15.310564014530270 14.814348023143937
H 12.995467099484065 17.147481241533175 16.338581215859321
H 13.974318135795423 19.522712619611543 16.432761929038584
H 16.155088028319842 20.124994882410029 15.127166255055949
H 17.166524424909884 18.496326353973274 13.406237590343723
H 29.854868664694447 18.130269437054590 21.397591168620004
H 28.908942737468198 18.046002747409979 19.681478201566073
H 30.438348406051162 19.942624480825227 18.772115558250590
H 31.316482193871728 18.271455336390936 19.113660935991746
H 30.318605403390645 21.295705249722335 21.479273101506955
H 32.137668500794831 22.477129245691817 23.048893471887325
H 34.645720807409532 21.658937508043760 22.991809289018430
H 35.340612947145750 19.634465876181064 21.402407354704970
H 33.495661364671875 18.506478384705385 19.827069079831077
H 25.968255171917992 18.331782042587701 9.614484749978420
H 24.678951637247771 19.515311041095103 10.335935275174087
H 27.354501263379692 20.402816798710386 8.682544750327894
H 25.498054674327097 20.627181301468109 8.180071483692860
H 28.404361865311628 21.743284168829994 10.512682434887784
H 28.270181833920422 23.785457915034428 12.149918926777740
H 25.937403033335734 24.989655621032139 12.521673004210264
H 23.744801450834871 24.061557967625902 11.393956010468004
H 23.932872189223772 22.086660206879241 9.665350172196961
H 22.064521002159058 19.126775075383144 12.817732697059551
H 23.446068071156564 19.792779682887097 11.742298454392436
H 20.574454382192847 18.623738844821364 10.797926371515461
H 21.337441472314747 20.346871191514502 10.407194191977052
H 23.402015820291741 20.441729204824139 8.801639243005159
H 24.924973264596431 19.196319031592576 6.975240889771311
H 24.669035564662583 16.593992033837086 6.617883940400293
H 22.829349088900010 15.242825163599663 7.999547469159904
H 21.317201999663553 16.552249322357280 9.751569641536216
H 26.611332897428685 22.176767600957195 14.623474571639555
H 26.712732054154198 22.884978141662920 16.376136469751369
H 27.905568819039591 24.327967046530720 14.392959527505321
H 28.958128840062962 24.013120685921773 15.929821023900150
H 31.253713475434232 23.427373049004238 15.340028423263821
H 32.959210369764300 21.823393485598384 14.047821284865552
H 32.036309677703677 20.080935178226472 12.264195967266531
H 29.384800560348147 19.981580303982884 11.787434470840431
H 27.812231426967763 21.618194713149869 13.009251273068223
H 16.013316325801966 16.211449750641183 23.049825164910541
H 15.742346943480044 14.692532405055049 25.146824630127934
H 12.031807053870670 17.153822743869004 26.297880378091069
H 12.392306657087738 19.876869572412829 24.152831362441301
H 11.150159298976234 18.676906925404715 24.928080373050758
H 11.453277350969492 18.659276227360625 23.084809299210086
H 14.550568591290588 14.268469157691710 27.325425822572619
H 12.653917981354088 14.173787865762499 26.849829053222145
H 13.265211323649245 15.588952952404549 28.038527351895947
H 15.378956992722101 21.145021727547338 13.884566909544789
H 16.938408415308935 26.110062496449334 13.486807288132276
H 18.903703683099092 25.196427098378670 15.197394092094180
H 18.029731298855015 24.526041435053806 16.758502697077912
H 17.382348167369660 26.100311169495818 15.913229695627694
H 14.749035763142258 22.476608803750494 11.829415639984193
H 16.092484449586458 24.559298914344463 10.336250252972475
H 15.972501236764590 26.254539981071563 11.312977122193610
H 14.352427735694912 25.197287581919383 10.972953763751903
H 13.292249894892320 31.732619936976018 22.623626575350109
H 14.598865266490572 30.580655188567441 23.511014076880894
H 12.962240308682409 29.867899789308574 22.837701222915982
H 13.241935003300162 32.009660588766032 20.250537362985284
H 15.189651302226935 31.925803099687304 16.800667022883104
H 14.483385374496285 33.206104886898451 18.096502301527785
H 13.281774720625515 31.915463030190121 17.296780033057907
H 16.464303164207521 30.061245400858223 17.666021314827891
H 17.243828200864609 28.705453309441214 19.744712991720775
H 8.651349761961500 29.443563614174547 22.406035901507622
H 6.127222419993388 29.636190226514959 21.636904887547328
H 5.062428215845959 29.667966628026040 17.880954636482933
H 4.531987066987190 29.842914818250478 19.755753986027713
H 4.804822516711262 28.077006489375734 18.973450468284167
H 7.456371671743243 28.627392125233484 17.396526774009267
H 9.615521242839467 28.331907104167247 16.791243290791510
H 10.650179483521484 27.431741407991279 18.098591746399048
H 11.000895258967716 29.289791431076249 17.721963930029357
H 16.388547253701269 18.968238378293666 28.511590281463249
H 17.474159288043563 19.161927524281243 30.864232168489259
H 17.528661834943847 20.256862094778565 33.044091857046716
H 16.385069262595199 21.766776530267439 33.469050271592202
H 18.121316673114539 22.057663629822944 32.627599041637765
H 15.497240333331401 23.248664085736973 31.364854208413686
H 13.439631723756941 23.010732885853482 28.172382472095645
H 15.076951510826268 23.959138940808675 28.318926910252028
H 13.845099135971685 23.967746444728807 29.777341882928429
H 16.929482591073761 19.486306906892327 17.144805747204703
H 16.665622164229358 17.289483085580205 15.872245387131349
H 15.803964204084457 14.867687134549106 16.141388390128238
H 16.472776875183289 13.786392916016295 17.618173462026327
H 17.673329188439929 14.420453355727037 16.246583749230020
H 17.380589775103900 14.990446151618043 19.705089538838443
H 18.769175252097615 17.578644147954474 21.628227449846197
H 18.089377137589814 15.850309291854519 21.455397552530673
H 16.941492647151371 17.278352804950696 21.970521452981057
H 18.253681868515756 19.796122472785623 27.216061286222910
H 19.571753385848400 19.046050032107779 28.356092803894924
H 18.137528061510274 17.928378100785057 27.677856316458975
H 21.584244483331190 18.094603025152225 27.486805522634132
H 22.297203510946783 18.040463188868767 23.014045647731759
H 24.111170073746489 17.218081551035684 24.611029857568397
H 22.988658937572524 15.997798644259969 25.555155105797329
H 23.827747957962231 17.392224323776080 26.566204581310860
H 19.805770709896787 18.609013085018525 22.628245282221364
H 14.011337293366291 27.085921114004879 24.717497925008139
H 14.568515136726743 28.884786362196138 24.631846571605013
H 12.960038828554795 28.298012285086713 23.791895828052574
H 14.638185762590270 29.729488286648888 26.729737435665399
H 11.226366572355936 29.177016004711341 29.697507810671141
H 14.629194248941854 31.043906204082791 28.791653478690474
H 14.254212996412944 29.705487480035913 30.144681074741936
H 12.999481884927851 31.175483314073073 29.878988401991723
H 9.938083762652139 27.719626366760512 27.911750710780350
H 12.167271722707897 28.340803417575248 16.665655179888187
H 11.271557510083177 27.861993887192348 14.274644526136646
H 13.785069049988653 24.034911436758417 14.156949956146265
H 12.022572980879048 24.393179667969918 12.492138653814022
H 12.594610653339595 26.113740398151446 11.780060590275795
H 10.809801067275318 25.924421816358169 12.551112308445257
H 14.747117120295583 23.327042247137950 15.984286000688652
H 14.194435104348328 23.894840627230433 17.709871831384536
H 15.810027827520008 24.545136388037125 16.978232139914699
H 11.788987289554880 24.934015961107885 19.144867082413729
H 11.737464762700908 24.852855235844974 16.507852800252575
H 7.460835765504943 26.367431272645920 16.596733908698219
H 10.428586915811740 24.853565035519161 14.464574818858491
H 9.552617795550995 26.575096270016346 14.403993534326476
H 8.470598576539103 24.967011558351043 14.570880518455247
H 6.299974000229892 26.720333380569322 18.673376562361856
H 7.364078916852692 27.070998212902353 20.264323671199914
H 6.696252551191992 25.287726244694060 19.924289730202936
H 11.063105687888658 16.125408658580440 17.962990147430105
H 9.531911677167431 17.814356820736720 17.438205975413357
H 10.442254079386469 19.237595337313412 16.465564009526457
H 9.698190186771551 19.562686246601771 18.199342998361921
H 12.815836929836236 14.367250552643823 17.801796336447801
H 13.811124269031795 14.433234111116342 19.487465677588521
H 14.683825447630705 14.937738570441104 17.833430888130369
H 15.360533304108003 17.069115566516242 19.117572377497112
H 14.812711875836488 19.559698863693772 19.105311135058322
H 16.622370465268059 25.638307336912007 18.704927692835994
H 16.498325924477658 27.718847683408157 17.270559851159415
H 20.772500387029567 28.783872494091856 18.515932009225164
H 17.785808233690926 29.428065145205139 16.018710122206006
H 19.725494663651070 29.449071811880472 16.194415230040153
H 18.637529282684017 30.622988562580545 17.312785875495592
H 22.003100880713404 27.360401839769917 19.964215146187868
H 21.562872488876376 25.501880291854185 19.743612298915373
H 21.016816127990854 26.371611259771356 21.338214314647157
H 22.528530083759776 25.480238725783984 24.443575189545179
H 21.255679728405461 24.482826524583295 25.519986892932373
H 22.694204718458920 25.428074119589020 26.379381853706775
H 22.033437901948300 27.273646813715768 27.617183674318685
H 18.995082367057471 30.242590498119004 26.068200951383712
H 20.455108170065280 30.890250221628381 27.864595526909081
H 21.578020148091003 29.583389194350559 28.816568583599398
H 19.626020298479006 29.467184096974716 28.900690919321679
H 18.455369349505446 28.659176700774935 24.013543212347365
H 8.169934906469733 19.625782214453871 23.346005842239201
H 8.236262142897939 17.071060531323937 24.044052751790243
H 8.718006173721454 15.337290369227512 25.784806520917748
H 9.364545609244471 15.862367772296482 27.559214412464634
H 10.634751389772727 15.577444622041771 26.109551279775914
H 10.718228948905836 18.168543187636537 27.689971984660321
H 11.354828163759988 21.757493216089667 26.718891859727378
H 12.262930038187939 20.124720848076759 26.453861282699336
H 11.442634297141707 20.407888090740542 28.106556749271519
H 16.483206038878723 27.536401088135623 24.900755710978459
H 17.202596734039190 30.006583314075968 25.167338855584660
H 17.920717390566153 31.857404015459576 26.731109028579823
H 18.287575418767009 31.317175002184026 28.566426181756103
H 16.591844681177051 32.153468164772093 28.107378122875016
H 16.316927888790538 29.742580208361851 29.615996326802208
H 14.892168343898865 26.144805727085714 29.469139546162840
H 13.732581595463609 27.685600193539511 29.375839218525403
H 15.269681833554074 27.710708763671619 30.537246063741854
H 21.171896150108267 20.471956243829631 24.614493875326175
H 22.186180790349564 20.398963391658970 26.225828848151263
H 22.032511038906055 22.073922356983228 25.284327870155263
H 21.280209191817281 21.091168968284912 28.361855083994062
H 20.266422182641570 22.258255470891140 30.523935709616349
H 19.002936477616341 20.774493492330759 30.661456326673402
H 18.318848392468826 22.556748290387777 30.555865666817024
H 16.942347292829972 22.402571331286790 28.631222320954539
H 16.729266082153064 22.153469265711760 26.019593110414441
H 7.903325689126436 16.408571932500919 19.264891781857092
H 9.004984551158843 16.303050668235237 21.453924808575891
H 9.540052445844616 17.887020477694737 22.437797047593605
H 10.612556754617293 17.225740717937221 20.977991315120306
H 5.862000795748587 18.314131974709372 16.501936141557490
H 6.169985541668364 16.580873085833648 17.371948575009398
H 7.561072617688699 17.384644024597161 16.275283788916173
H 6.720443550040468 20.457759809125221 17.515421193606624
H 8.119498508503369 21.652158738606289 19.393547088395593
H 10.856253930838291 22.154894323458382 28.613289961757854
H 12.383903974958967 20.709731024743018 30.198106496176003
H 12.800678843531887 21.062493715161501 33.352498585797321
H 14.350530749156784 22.238994600457659 33.210499228448747
H 14.184309283003937 20.739431190905947 31.983384610072768
H 13.250598154679361 24.424294449603089 32.657037519468346
H 11.866987788810958 26.791886324274014 29.909359745098172
H 13.157441273914287 26.570428622585421 31.336091628883892
H 11.256862931806229 26.688855211454367 31.719158542071085
